# Genome-Wide Identification of NBS-Encoding Resistance Genes in Sunflower (*Helianthus annuus* L.)

**DOI:** 10.3390/genes9080384

**Published:** 2018-07-30

**Authors:** Surendra Neupane, Ethan J. Andersen, Achal Neupane, Madhav P. Nepal

**Affiliations:** Department of Biology and Microbiology, South Dakota State University, Brookings, SD 57007, USA; surendra.neupane@sdstate.edu (S.N.); ethan.andersen@sdstate.edu (E.J.A.); achal.neupane@sdstate.edu (A.N.)

**Keywords:** coiled coil, disease resistance, nucleotide binding site encoding genes, gene clustering, plant defense, resistance pathways, resistance to powdery mildew 8, R genes, sunflower, synteny

## Abstract

Nucleotide Binding Site—Leucine-Rich Repeat (NBS-LRR) genes encode disease resistance proteins involved in plants’ defense against their pathogens. Although sunflower is affected by many diseases, only a few molecular details have been uncovered regarding pathogenesis and resistance mechanisms. Recent availability of sunflower whole genome sequences in publicly accessible databases allowed us to accomplish a genome-wide identification of Toll-interleukin-1 receptor-like Nucleotide-binding site Leucine-rich repeat (TNL), Coiled Coil (CC)-NBS-LRR (CNL), Resistance to powdery mildew8 (RPW8)-NBS-LRR (RNL) and NBS-LRR (NL) protein encoding genes. Hidden Markov Model (HMM) profiling of 52,243 putative protein sequences from sunflower resulted in 352 NBS-encoding genes, among which 100 genes belong to CNL group including 64 genes with RX_CC like domain, 77 to TNL, 13 to RNL, and 162 belong to NL group. We also identified signal peptides and nuclear localization signals present in the identified genes and their homologs. We found that NBS genes were located on all chromosomes and formed 75 gene clusters, one-third of which were located on chromosome 13. Phylogenetic analyses between sunflower and *Arabidopsis* NBS genes revealed a clade-specific nesting pattern in CNLs, with RNLs nested in the CNL-A clade, and species-specific nesting pattern for TNLs. Surprisingly, we found a moderate bootstrap support (BS = 50%) for CNL-A clade being nested within TNL clade making both the CNL and TNL clades paraphyletic. *Arabidopsis* and sunflower showed 87 syntenic blocks with 1049 high synteny hits between chromosome 5 of *Arabidopsis* and chromosome 6 of sunflower. Expression data revealed functional divergence of the NBS genes with basal level tissue-specific expression. This study represents the first genome-wide identification of NBS genes in sunflower paving avenues for functional characterization and potential crop improvement.

## 1. Introduction

Plants employ different gene families in signaling networks in response to numerous biotic and abiotic stresses [1]. In order to deal with these stresses, during the course of evolution, plants have developed multifaceted processes to recognize the stress stimuli, transfer them to the plant’s own message(s) and complete the signal transduction pathways [2,3]. In response to the stresses due to pathogens, plants have developed race-specific and race non-specific resistance, known as qualitative and quantitative resistance, respectively [4]. Plants recruit proteins encoded by disease resistance (R) genes that recognize or interact with specific pathogen avirulence (*avr*) gene products [5] or effector molecules triggering a downstream signaling in resistance pathways [6,7]. Various models, such as Zig-zag and multicomponent models, propose a dynamic relationship between a host and its pathogen and explain how incompatible interactions between the hosts and pathogens lead to a selection of new R genes in response to co-evolutionary pressure due to pathogen races [6,8]. Host R genes can vary within a species, and their variation is correlated with that of the corresponding pathogen effectors [9]. For example, host polymorphic to R genes is found to provide partial resistance against pathogens [10]. Such partial resistance accumulates throughout the plant development and eventually provide quantitative resistance in the form of broad spectrum resistance [10]. Identification of R genes and their pathogen effectors is essential for understanding host–pathogen interactions and disease resistance pathways in order to develop durable resistance in crop species.

The Pathogen Recognition Genes database (PRGdb, http://prgdb.org) listed 153 R genes that have been cloned and characterized, and 177,072 annotated candidate Pathogen Receptor Genes (PRGs) [11]. These R genes encode mostly nucleotide binding site (NBS) leucine-rich repeat (LRR) proteins and have been classified into categories based upon the domains and motifs organization in the proteins [12,13]. Most commonly recognized categories are Toll-interleukin-1 receptor-like-NBS-LRR (TNL), Coiled-Coil-NBS-LRR (CNL), and Resistance to powdery mildew8 (RPW8)-NBS-LRR (RNL) [13,14]. All TNL, CNL and RNL genes are present in dicots, whereas TNL genes are absent in monocots [14,15]. Analysis of NBS genes in Fabaceae and Brassicaceae revealed that CNLs and RNLs diverged prior to divergence of Rosid I and Rosid II lineages of Angiosperms, and, in both plant families, the two clades are sister to each other [15,16]. The NBS domain, also known as NB-ARC where ARC stands for APAF1 (apoptotic protease-activating factor-1), R genes, and CED4 (*Caenorhabditis elegans* death-4 protein), hydrolyzes ATP to induce the conformational change in R proteins by acting as the nucleotide binding pocket [17]. The NBS domain mainly consists of P-loop, Kinase-2, RNBS A, GLPL and MHDL motifs [14]. The LRR domains at the C-terminus help activate or deactivate the defense signaling by interacting with the NBS domain in the presence or absence of pathogen effectors, respectively [18]. A diverse number of NBS genes have been reported in various plant species since the first study in *Arabidopsis thaliana* was published in 2003 [14]. With the increasing availability of plant genome sequences, R gene proteins have been identified in many plant species, such as *A. thaliana* [5,14]; *Vaccinium* spp. [19]; *Amborella trichopoda*, *Musa acuminata*, *Phyllostachys heterocycla*, *Capsicum annuum*, and *Sesamum indicum* by Shao et al. 2016 [13]; *Cicer arietinum* [20]; *Glycine max* [21,22,23,24]; *Oryza sativa* [25,26]; *Medicago truncatula* [27]; *Vitis vinifera* and *Populus trichocarpa* by Yang et al. 2008 [28]; *Solanum tuberosum* [29]; *Brassica rapa* and *Brassica oleracea* by Zhang et al. 2016 [30]; *Hordeum vulgare* [31]; *Setaria italica* [32]; *Theobroma cacao* [5]; *Populus trichocarpa* [5]; *V. vinifera* [5]; *Cucumis sativus* [33]; *Phaseolus vulgaris* [16,24], *Lotus japonicas*, *Cajanus cajan*, *Glycine soja* by Zheng et al. 2016 [16], *Gossypium arboretum* [34], etc. A recent study by Li et al. 2016 [35] has identified NBS-encoding genes as well as receptor-like protein kinases (RLKs) and receptor-like proteins (RLPs), collectively called as Resistance Gene Analogs (RGAs), for 50 plant genomes using a RGAugury pipeline.

According to a report by Food and Agriculture Organization (FAO) in 2010 (http://www.fao.org), domesticated *Helianthus annuus* L. (Family Asteraceae), is the fourth most important oilseed crop in the world. Since sunflower has the capacity to maintain stable yields in different environmental conditions such as drought, it has been a model crop species for studying climate change adaptation [36]. The study on diversity analysis of 128 expressed sequenced tag (EST)-based microsatellites in wild *H. annuus* has provided insights into the ability to adapt salt and drought stress and selective sweeps revealing transcription factors as the major group of genes involved in those processes [36]. In addition, studies on wild and cultivated relatives of sunflower on disease resistance [37] and oil content [38] aspects have played great roles in understanding the genetic background for these traits. However, many fungal diseases like charcoal rot (*Macrophomina phseolina*), downy mildew (*Plasmopara halstedii*), *Fusarium* rot and stem rots (*Fusarium* sp.), phoma black stem (*Phoma macdonaldii*), phomopsis stem canker (*Diaporthe helianthi*, *D. gulyae*), *Sclerotinia* mid and basal stem rot (*Sclerotinia scelerotiorum*), *Verticillium* wilt (*Verticillium dahlia*), leaf blight (*Alternariaster helianthi*), leaf spot (*Pseudomonas syringae* pv. *helianthi*), powdery mildew (*Erysiphe cichoracearum*), rust (*Puccinia helianthi*) and many others have caused crop damage resulting in the loss of yield and oil content [39].

Previously, various studies have contributed their findings about the NBS group of R genes in sunflower [40,41,42,43]. Plocik et al. 2004 [40] identified nine sunflower resistance gene candidates with coiled-coil (CC) domains in the N-terminal region using degenerate primer sets. Sunflower showed diverse structures in CC subfamily, while lettuce and chicory, closely related species, showed high similarity in structure. Radwan et al. 2008 [42] used degenerate primers to identify 630 NBS-LRR homologs in wild species of sunflower (*Helianthus annuus*, *Helianthus argophyllus*, *Helianthus deserticola*, *Helianthus paradoxus*, and *Helianthus tuberosus)*. In addition, Radwan et al. 2004 [43] isolated R gene analogs belonging to the CNL class of R genes from the inbred sunflower line QIR8 containing *Pl8I* locus against *P. halstedii*, which causes downy mildew. Later, Hewezi et al. 2006 [41] cloned partial antisense *PLFOR48*, which showed homology to the TNL family, in mildew resistant sunflower line, RHA 266 and *Nicotiana tabacum* L. The recent availability of the *H. annuus* genome [44] has now made it possible for studying the diversity and evolution of gene families in sunflower. The main objectives of this research were to conduct a genome-wide search for *H. annuus* NBS genes and analyze their genomic structure and functions. A proper identification of the R genes is crucial to elucidate their roles against various diseases in sunflower.

## 2. Materials and Methods

### 2.1. Retrieval and Identification of Sunflower NBS-Encoding Genes

The genome of sunflower (INRA inbred genotype XRQ described in [44]; *H. annuus* r1.2) was accessed from the sunflower genome database (https://www.sunflowergenome.org) as well as Phytozome (https://phytozome.jgi.doe.gov). The sunflower genome is 3.6 gigabases and its genes distributed over 17 chromosomes encode 52,243 proteins (https://phytozome.jgi.doe.gov). *A. thaliana* TNL and CNL (= nonTNL or nTNL) protein sequences were used as reference for the identification of NBS-LRR proteins in sunflower, and were obtained from http://niblrrs.ucdavis.edu. The multiple sequence alignment file of these reference sequences in Stockholm format were employed in hmmbuild and hmmsearch for HMM profiling using the program HMMER version 3.1b2 [45] at a cut-off value of 0.01. InterProScan Version 5.27 (EMBL-European Bioinformatics Institute, UK) [46] and Pfam ID [47] and PROSITE ID (http://prosite.expasy.org/) were used to search for the conserved domains. The proteins with PfamID of TIR (PF01582), NBS (PF00931), RPW8 (PF05659), CC and LRR domains with ‘LxxLxxLxx’ signatures were selected to determine the NBS proteins in sunflower. Further verification of the CC domains at the N-terminus was carried out using the MARCOIL server [48] with a 9FAM matrix having probability between 0.4–1. Multiple expectation maximization for motif elicitation (MEME) [49] analysis was performed to confirm the presence of P-loop, Kinase-2, GLPL, MHDL, RNBS A, RNBS B, RNBS C, and RNBS D motifs in the NBS domain, TIR1, TIR2, TIR3 motifs in TIR domain and RPW8 motifs in RPW8 domains. A set of parameters used in MEME analysis included maxsize: 100,000, mod: zoops, nmotifs: 20, minw: 6, and maxw: 50 to 25. Subcellular localization of the putative NBS genes were analyzed using TargetP 1.1 [50]. The program NLStradamus [51] was used to examine nuclear localization signals (NLS) in identified NBS genes of sunflower using a two-state HMM static model with Viterbi and posterior prediction methods (with 0.5 cut-off).

### 2.2. Phylogenetic Tree Construction

The NBS protein sequences from *A. thaliana* and *H. annuus* were aligned using CLUSTAL W [52] and MUSCLE [53] integrated in the program Geneious [54]. Phylogenetic analysis of the aligned data matrix was performed using Maximum Likelihood (ML) method (1000 replicates) in the program MEGA Version 7.0.14 [55]. The phylogenetic analysis employed the best evolutionary model (resulted from the ModelTest analysis using MEGA7) JTT + G + I (Jones–Taylor–Thornton with γ distribution and invariant sites), and *Streptomyces coelicolor* accession P25941 as an outgroup [14]. Additional phylogenetic trees of the NBS domains of predicted TNL and CNL proteins of sunflower and all reference proteins obtained from http://prgdb.crg.eu were reconstructed using the methods and models described above. Thus, the obtained Newick format of phylogenetic trees were employed in the Interactive tree of life (iTOL) Version 3 (Biobyte solutions GmbH, Bothestr, Germany) for their visual enhancement [56].

### 2.3. Chromosomal Locations, Clustering and Gene Structure

All 17 chromosome sequences of *H. annuus* were obtained from https://www.sunflowergenome.org and uploaded in the program Geneious [54]. The chromosome locations of the respective gene families were visualized using an annotation file in Generic Feature Format (GFF). The NBS gene locations, NBS types and clustering were visualized on their respective chromosomes. Gene clustering of the NBS genes was carried out following Jupe et al. 2012 [57], using two major criteria: (a) distance between two NBS genes is less than 200 kb, and (b) presence of no more than eight annotated non-NBS sequences between two consecutive NBS sequences. The exon-intron distribution pattern was obtained by the Gene Structure Display Server (http://gsds.cbi.pku.edu.cn).

### 2.4. K_a_/K_s_ and Syntenic Analysis

Coding sequences (CDS) of the NBS genes were used in calculating nonsynonymous substitutions per nonsynonymous site (*K_a_*) and synonymous substitutions per synonymous site (*K_s_*) in the program DnaSP 6.11.01 [58]. Syntenic map of the NBS genes of *H. annuus* and *A. thaliana* was created using SyMAP Version 4.2 (Arizona Genomics Computational Lab, Tucson, AZ, USA) [59], executed within South Dakota State University’s High-Performance Computing Cluster. Whole chromosome sequences and gene annotation files were used as input files for syntenic mapping of *H. annuus* and *A. thaliana*.

### 2.5. Gene Homology and Expression Analysis

Putative homologs of the predicted sunflower NBS genes were accessed using BLAST tool available in http://prgdb.org with reference genes of PRGdb and a cutoff E-value of 0.01. The filtering included sequences with E-values less than 0.01 and identity percentage of greater than 50%. Expression profiles of the putative NBS genes were downloaded from https://www.sunflowergenome.org. A heatmap was generated using deseq normalized data through the MeV package, available at http://mev.tm4.org/ [60]. The heatmap clustering was performed based on Euclidean distance under 1000 iterations using the K-means Clustering Method. The clustering classification used these categories: moderate to minimal expression, minimal expression to no expression, and no expression at all.

## 3. Results

### 3.1. Diversity of the NBS-Encoding Genes in Sunflower

The HMM analysis of all sunflower protein-coding genes using the reference sequences of *A. thaliana* resulted in 485 NBS proteins, using a filtering threshold expectation value of 0.01. These sequences were further annotated with InterProscan, and evaluated for the presence of NBS domains in each sequence. After a careful examination, 352 protein sequences were confirmed to have an NBS domain. Among these, 100 genes belonging to CNL group (after verification using MARCOIL server omitted ten false positives), 77 to TNL, 13 to RNL group, as well as 162 genes possess neither CC nor TIR domains thus classified as an NL group. Among 100 CNL types, 64 possesses a CC domain similar to *S. tuberosum* disease resistance protein (Rx). Furthermore, Leucine-rich repeats (LxxLxxLxx signatures) were examined to classify CNLs, TNLs, RNLs and NLs into their subgroups. Following the classification of NBS-encoding genes in *Brassica* species and *A. thaliana* [5], the NBS genes were classified into: CC-NBS-LRR (CNL), CC-NBS (CN), CC-NBS-NBS-LRR (CNNL), CC-NBS-NBS (CNN), RPW8-NBS-LRR (RNL), RPW8-NBS (RN), RPW8-CC-LRR (RCL), TIR-NBS-LRR (TNL), TIR-NBS (TN), TIR-TIR-NBS-LRR (TTNL), TIR-NBS-LRR-TIR-NBS-LRR (TNLTNL), TIR-CC-NBS-LRR (CTNL), TIR-CC-NBS (CTN), NBS (N), NBS-LRR (NL), NBS-NBS (NN), and NBS-NBS-LRR (NNL) (see Table 1, Figures S1–S4). The LxxLxxLxx (=LRRs) signatures were present in 97 (out of 100) CNL genes with their LRRs ranging from two to 22, 12 (of 13) RNL genes with one to eight LRRs, 55 (of 77) TNL genes with two to 26 LRRs, and 131 (of 162) NL genes with two to 30 LRRs. Among them, HanXRQChr02g0052061, a TNL protein sequence contained a unique Kelch motif sequence (PF01344). TargetP analysis showed that 20 NBS proteins were predicted to localize to the chloroplast, 14 to mitochondria, 80 enter the secretory pathway, and 238 were predicted to enter other subcellular locations other than mitochondria or the chloroplast (Table S1). Thirteen CNLs, seven TNLs, one RNL, and eight NLs were identified to contain a putative NLS using NLStradamus (Table S2).

Three major signature motifs: P-loop, Kinase-2, and GLPL of the NBS domain of disease resistance proteins were present in 57 out of 100 CNLs, 69 out of 77 TNLs, all 13 RNLs and 58 out of 162 NLs (Supplementary File S1, Figures S5–S7). Other important motifs RNBS A, RNBS B, RNBS C and RNBS D, and MHDL were also present in the NBS proteins (Tables S3–S5). Motifs TIR1, TIR2, TIR3, and TIR4 varied in number across the TNL genes: among the 77 TNLs, 76 had TIR1, 76 had TIR2, 75 had TIR3 and 76 had TIR4 motifs. Only two TNLs (HanXRQChr05g0136351 and HanXRQChr06g0184071) did not have all four TIR motifs. Of the 100 CNLs, 81 had the characteristic conserved amino acid sequence ’DDVW’ in the Kinase-2 motif. Remaining CNLs had either Isoleucine (I), Methionine (M), or Leucine (L) in the place of Valine (V) amino acid in the sequence ‘DDVW’. Of the 77 TNLs, 50 shared the characteristic ‘DDVD’ amino acid sequence in the Kinase-2 motif. Of the 162 NLs, 83 had ‘DDVW’ and 18 had ‘DDVD’, hence classified as N_CC_ and N_TIR_ group of the NLs, respectively. All of the 13 RNLs had ‘DDVW’ sequence in the Kinase-2 motif except for HanXRQChr03g0067681 with ‘DDVR’ sequence. Another key characteristic found within the RNBS B motif was that the majority of the CNLs had ‘TSR’, TNLs had ‘TTRD’, and RNLs had ‘TSR’ residues. The sequence alignments illustrating all the conserved motifs of the CNLs, TNLs, and RNLs are presented in Supplementary File S2.

### 3.2. Gene Location, Clustering, K_a_/K_s_ Values and Structural Variation

**Figure 1:**
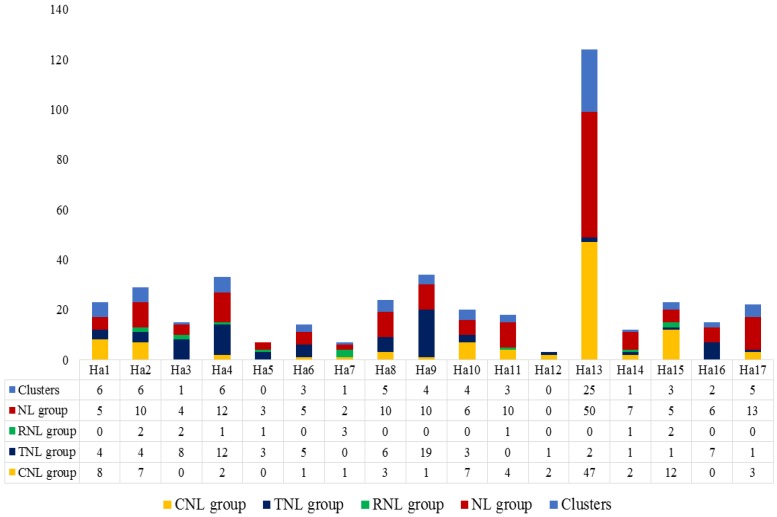
Chromosomal distribution (Ha1–Ha17) of the NBS genes and gene clusters in sunflower. Different NBS groups and gene clusters are color coded. CNL: Coiled-Coil-NBS-LRR; TNL: Toll-interleukin-1 receptor-like-NBS-LRR, RNL: Resistance to powdery mildew8 (RPW8)-NBS-LRR; NL: Nucleotide Binding Site—Leucine-Rich Repeat (NBS-LRR).

**Figure 2:**
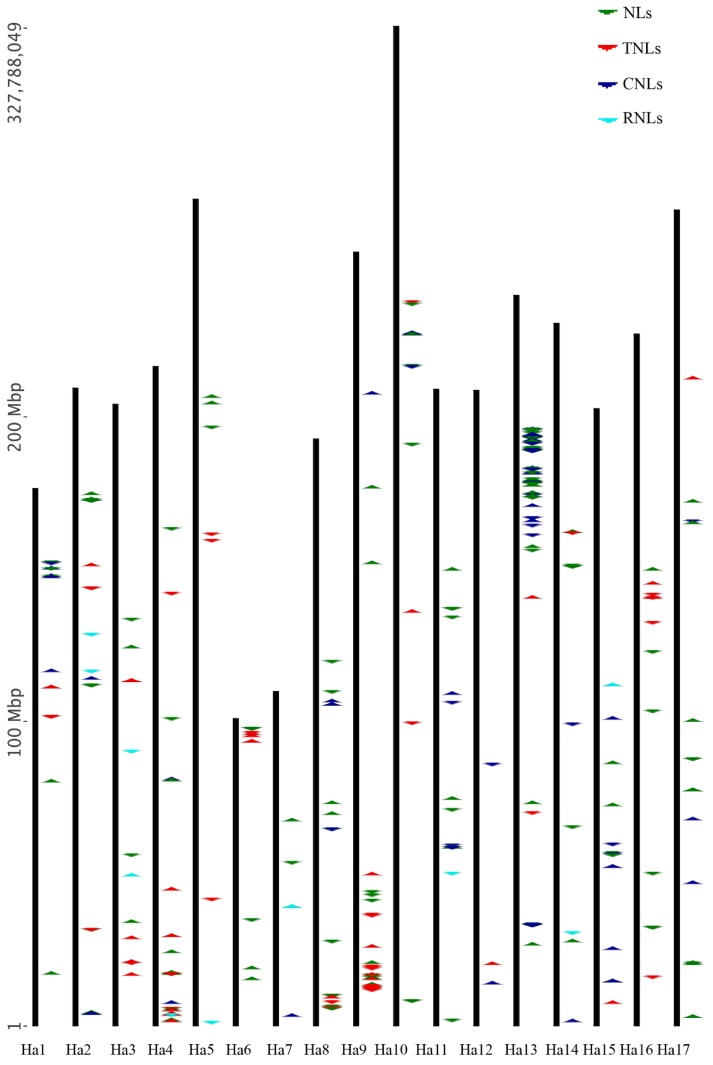
Chromosomal distribution of sunflower NBS gene clusters (*n* = 17). Each arrow color represents an NBS gene type and orientation, and the thick vertical line represents a chromosome.

The NBS genes are located on each of the chromosomes, with only four (*HanXRQChr00c0003g0570971*, *HanXRQChr00c0003g0570951*, *HanXRQChr00c0004g0571011*, and *HanXRQChr00c0037g0571241*) were not assigned to any chromosome (Figure S8). The number of the NBS genes located on each chromosome ranged from three (chromosome Ha12) to 99 (chromosome Ha13). Chromosomal distribution of the CNL, TNL, RNL, and NL genes and their clusters are shown in Figure 1. The CNL genes were absent in chromosomes Ha3, Ha5, and Ha16, whereas, TNL genes were absent in chromosomes Ha7 and Ha11. Most of the TNL genes were uniformly distributed across the chromosomes, whereas most of the CNL and NL genes were densely represented on chromosome Ha13 (approximately 28%). The smallest number of RNL genes (thirteen) were present in chromosomes Ha2, Ha3, Ha4, Ha5, Ha7, Ha11, Ha14, and Ha15 (see Figure 1). Among the 352 NBS genes, 200 (~57%) genes formed 75 clusters (4.4 clusters per chromosome and 2.7 genes per cluster) with chromosome Ha13 hosting 25 clusters of 73 genes (~37%; Table S6). The gene clusters were present in all chromosomes except for Ha5 and Ha12. Gene positions and clusters on chromosomes of *H. annuus* are shown in Figure 2. The average *K_a_/K_s_* values for the clades of CNLs, TNLs, and RNLs were 0.68, 0.89, and 0.31, respectively. The number of exons in the genes is shown in Table S1 and Figures S9–S12. The number of exons for CNLs, TNLs, RNLs, and NLs ranged from 1 to 11, 2 to 18, 4 to 9, and 1 to 19, respectively. In average CNLs, TNLs, RNLs, and NLs had 2.7, 6.1, 6.2, and 2.9 exons per gene, respectively.

### 3.3. Phylogenetic and Syntenic Analysis

**Figure 3:**
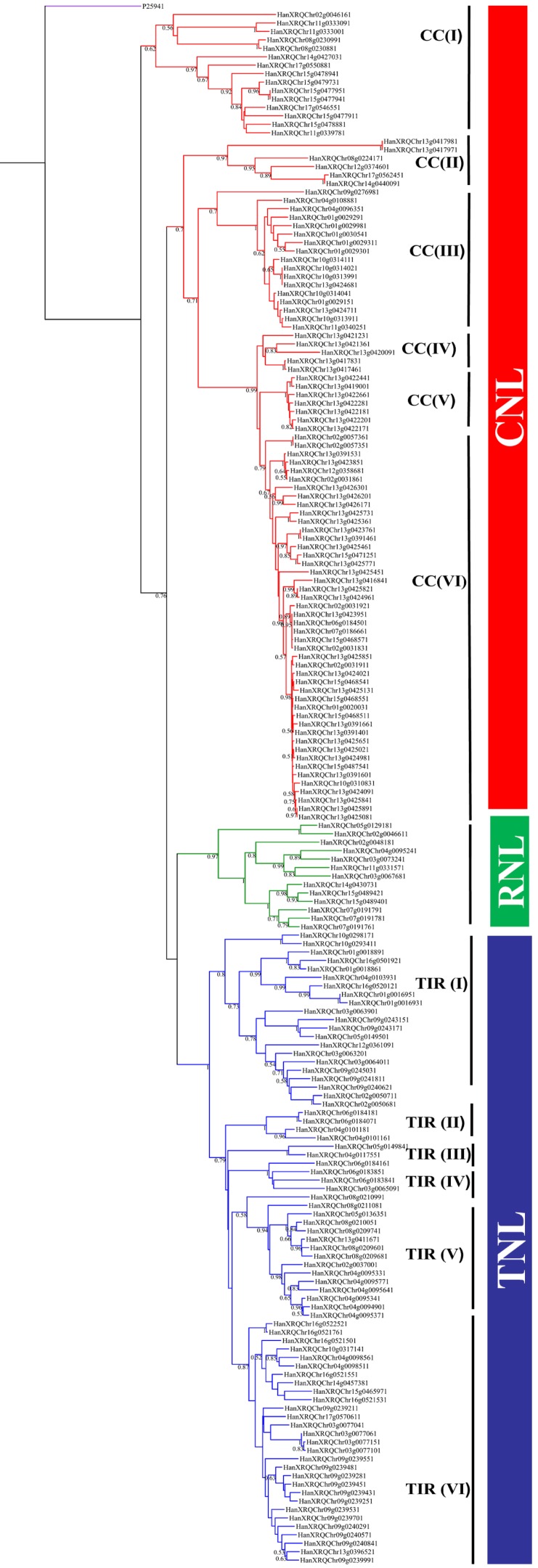
Maximum likelihood (ML) tree featuring NBS groups based on the conserved domains of the CNL, TNL, and RNL genes from *Helianthus annuus*. The ML tree was constructed using the JTT + G + I (Jones–Taylor–Thornton with γ distribution and invariant sites) model with 1000 bootstrap replicates. The ML tree was rooted using a *Streptomyces coelicolor* NBS containing protein, P25941, as an outgroup. The clades TNL (blue), CNL (red), and RNL (green) and outgroup (purple) are color-coded. Subclades are mentioned as TIR (I) to TIR (VI) and CC (I) to CC (VI).

**Figure 4:**
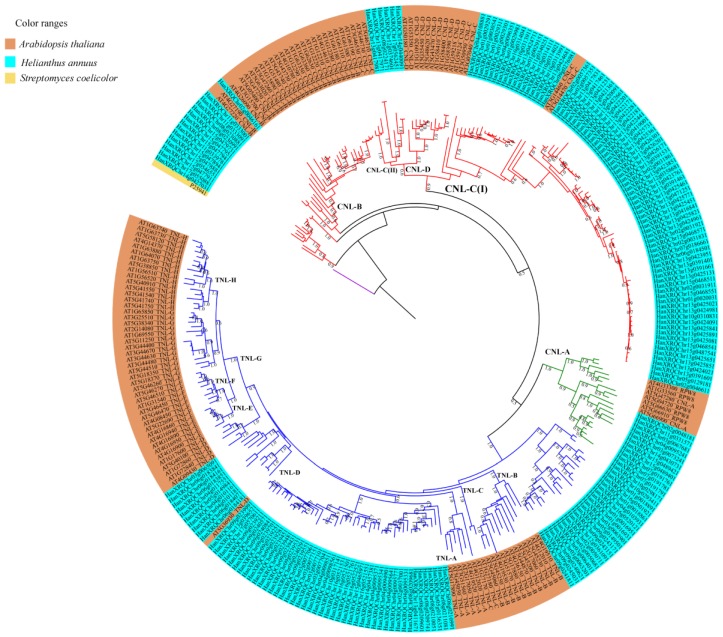
Maximum likelihood (ML) tree featuring NBS domain amino acid sequences of the CNL, TNL, and RNL genes from *Arabidopsis thaliana* (AT; orange) and *Helianthus annuus* (light blue). The ML tree was reconstructed using JTT + G + I (Jones–Taylor–Thornton with γ distribution and invariant sites) evolutionary model with 1000 bootstrap replicates. The ML tree was rooted using *Streptomyces coelicolor* NBS-containing protein, P25941, as an outgroup (yellow). The clades are color-coded: TNL in blue, CNL in red, RNL clade in green, and outgroup in purple. Subclades are labeled as CNL-A to CNL-D and TNL-A to TNL-H.

**Figure 5:**
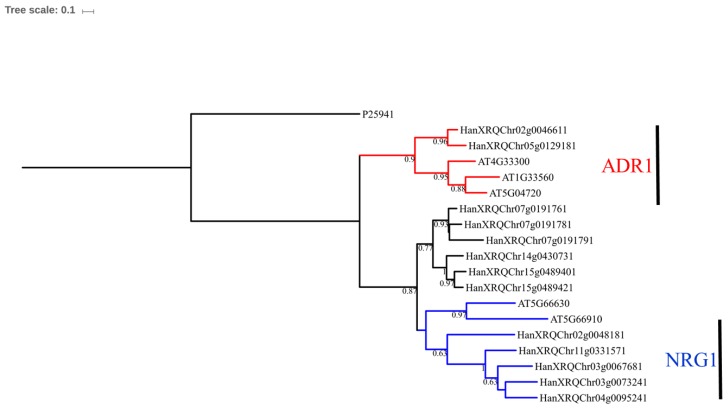
Phylogenetic relationships of RNL proteins in *Arabidopsis thaliana* and *Helianthus annuus*. The clades N-required gene 1 (*NRG1*) and activated disease resistance gene 1 (*ADR1*) are color-coded in blue and red, respectively. The tree was rooted using *Streptomyces coelicolor* NBS-containing protein, P25941, as an outgroup.

The data matrix with the NBS aligned sequences (NBS domain region is more conserved than remaining 5’ and 3’ regions) was used in phylogenetic analyses. Phylogenetic relationships among the sunflower NBS sequences are shown in Figure 3, and those of the sunflower and *Arabidopsis* NBS sequences are shown in Figure 4; each tree reveals distinct clades of CNLs, RNLs and TNLs. The RNL clade was surprisingly nested within the TNL clade. As shown in Figure 3, the CNLs and TNLs formed six subclades each. The TNL subclades are named TIR (I), TIR (II), TIR (III), TIR (IV), TIR (V), and TIR (VI), whereas CNL subclades are named CC (I), CC (II), CC (III), CC (IV), CC (V), and CC (VI). The phylogenetic tree reconstructed using sunflower and *Arabidopsis* NBS sequences revealed clade-specific nesting patterns in the CNL group (Figure 4). The nesting of all sunflower RNL genes within CNL-A clade (with *Arabidopsis* RPW8 genes) was strongly supported (bootstrap support = 96%). CNL-C (I) clade constituted six CNL genes (*HanXQRChr14g0440091*, *HanXQRChr17g0562451*, *HanXQRChr12g0374601*, *HanXQRChr08g0224171*, *HanXQRChr13g0417971*, and *HanXQRChr13g0417981*) with a weak support [bootstrap support (BS) = 57%]. CNL-C (I) clade, sister clade to CNL-C (II) and CNL-D constituted 79 genes. CNL-B clade constituted three genes (*HanXQRChr02g0046161*, *HanXQRChr11g0333001*, and *HanXQRChr11g0333091*). The remaining 12 genes did not belong to any clade of *Arabidopsis* CNL genes. The TNL group formed a species-specific clade, except ten genes that formed a small clade with AT5G36930, named TNL-D clade with strong bootstrap support of 100%. We found a moderate bootstrap support (BS = 50%) for CNL-A clade being nested within TNL clade making both the CNL and TNL clades paraphyletic. Another tree constructed using RNL genes of *A. thaliana* and *H. annuus* showed two distinct clades for two lineages: activated disease resistance gene 1 (*ADR1*) and N-required gene 1 (*NRG1*) (Figure 5). The Newick files related to phylogenetic trees in Figure 3, Figure 4 and Figure 5 are provided in Supplementary File S3. For the comparative study, all the manually curated TNL and CNL reference proteins obtained from http://prgdb.crg.eu were phylogenetically compared with sunflower TNL and CNL NBS proteins. The sunflower NBS proteins formed clades with various reference proteins such as Pi36, Pl8, Rps2, VAT, RPG1, Gro1.4, RY-1, and N proteins suggesting their homologs (Figure S13). The syntenic relationship between the *Arabidopsis*’s 119,146 kb genome and sunflower’s 3,641,596 kb genome showed 87 syntenic blocks with 1049 synteny hits. The chromosome 2 of *Arabidopsis* was highly syntenic to chromosome Ha1, Ha2, Ha3, and Ha15 chromosomes of sunflower. Similarly, the highest syntenic region was observed between chromosomes 5 of *Arabidopsis* and chromosome 6 of sunflower. The sunflower chromosomes Ha2, Ha5, Ha11, Ha13, Ha15, and Ha17 are least syntenic to any of the chromosome of *Arabidopsis*. The pericentromeric region of the sunflower chromosomes Ha3, Ha9, and Ha14 were highly syntenic to the chromosomes of *Arabidopsis*. The chromosome Ha13 that contains 99 NBS genes contains fragments from only chromosome 2 of *Arabidopsis* (Figure S14).

### 3.4. Homologs and Expression Analysis

**Figure 6:**
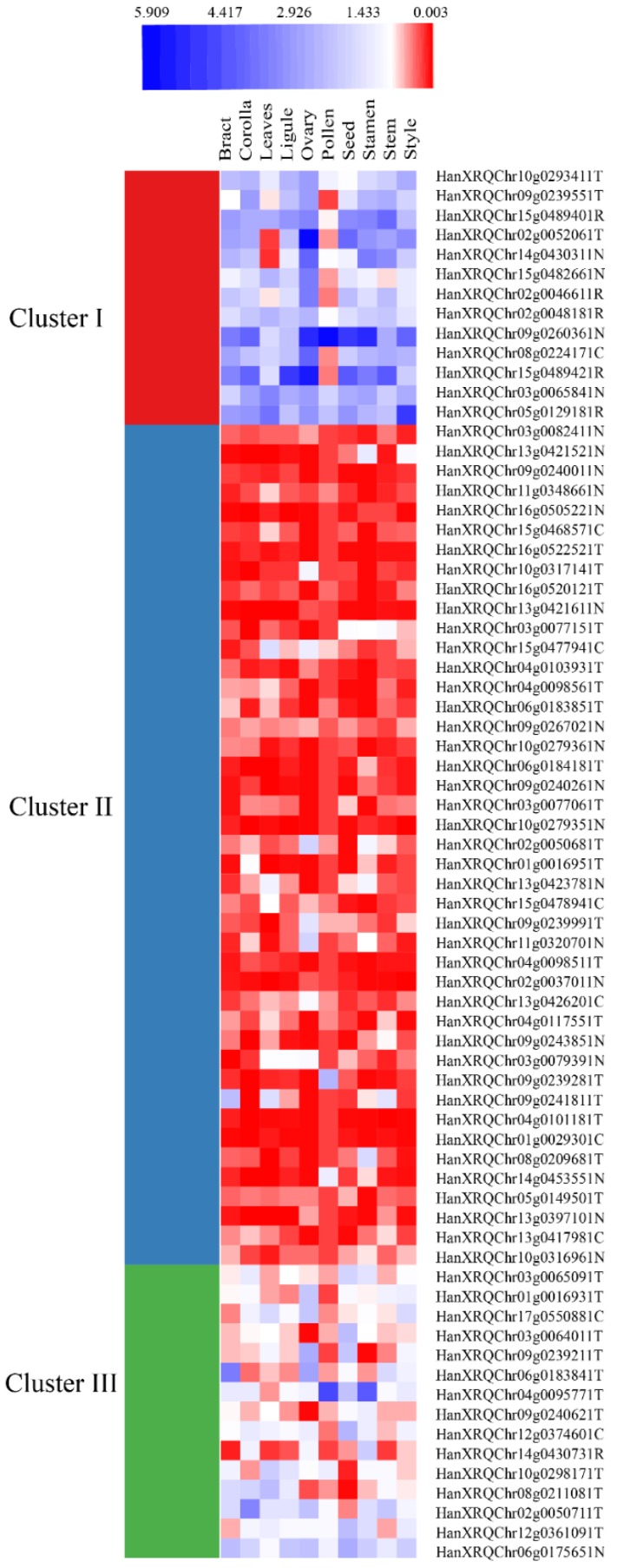
Expression profile of NBS genes from sunflower visualized as heatmap. The heatmap was generated using deseq normalized data for sunflower NBS genes expression in different tissues. K-means Clustering Method was employed for clustering (I, II and III). Gene IDs are followed by NBS type (C: CNLs; T: TNLs; N: NLs and R: RNLs).

The predicted 352 NBS proteins of sunflower showed homology, with identity greater than 50% and E-value less than 0.01, to 39 genes among 153 reference genes on the Plant Resistance Genes database (Table S7). Among them, 21 proteins showed greater than 70% identity to the *H. annuus* clone Ha-NTIR11g CC-NBS-LRR gene (*Pl8*). HanXRQChr13g0425411, HanXRQChr13g0425361, and HanXRQChr13g0425431 showed more than an 80% identity to the *Pl8* gene suggesting the probable homologs to that gene. HanXRQChr04g0123041, belonging to the NL group has shown homology to *Lycopersicon esculentum* EIX receptor 1 (*LeEIX1*), a gene that encodes receptor-like proteins (RLPs). Similarly, HanXRQChr17g0552491 showed homology to MLA10, HanXRQChr13g0420141 to N, HanXRQChr17g0552491 to both MLA12 and MLA13 and HanXRQChr17g0552491 to Sr33 protein with greater than 60% identity. Sunflower Genome Database with *H. annuus* r1 annotations was employed to obtain expression data for predicted NBS genes. We compared accessions of *H. annuus* r1.2 annotations to *H. annuus* r1 to obtain the expression data for NBS proteins. Since there were many duplicates for *H. annuus* r1.2 annotations, we used only the sequences with the unique names. The raw Read Per Kilobase Million) (RPKM) values of gene expression were downloaded separately. The expression values were from bract, corolla, leaves, ligule, ovary, pollen, seed, stamen and stem. Only expression data for 9 CNL type, 33 TNL type, 23 NL type and 6 RNL type genes were retrieved from the database and employed to generate heatmap after deseq normalization of the data using MeV package (Figure 6). Cluster I consists of 13 genes representing moderate to minimal expression, cluster II with 43 genes representing basal to no expression and cluster III with 15 genes representing minimal expression to basal expression (Figure S15).

## 4. Discussion

### 4.1. Diversity of NBS-Encoding Genes

Our findings on the NBS-encoding genes in this study is based on recently sequenced sunflower genome [44]. Previously, Gedil et al. 2001 [61] identified RGC fragments with the NBS domains and assigned to 11 groups among which Ha4W2A was linked to *Pl1*, a downy mildew resistance gene. Plocik et al. 2004 [40] identified nine unique NBS domain sequences using degenerate primers in sunflower and compared them to lettuce, chicory and *A. thaliana*. They concluded that NBS gene sequences of Asteraceae family are ancestral to the Brassicaceae family. Later, Radwan et al. 2008 [42] identified 118 and 95 NBS domain sequences in RHA373 and ANN-1811 germplasm of *H. annuus*, respectively. In this study, we identified 352 NBS-encoding genes that constitute 0.67% of the total predicted proteins in sunflower, which shows similarity to *M. truncatula* (~0.66%) [27]. This number is higher than that of *Arabidopsis* (~0.43%) [14], *C. sativus* (~0.21%) [33], *Carica papaya* (~0.21%) [62] and lower than that of *P. vulgaris* (~1.19%) [63], *Manihot esculenta* (~0.9%) [64], *V. vinifera* (~1.3%) [28], and *G. max* (~0.73%) [23,24]. We performed protein blast (BLASTp) analyses using 352 NBS domains of NBS-encoding genes identified in this study against a database with previously studied NBS domain sequences. The BLASTp analyses against a database comprised of sequences from Gedil et al. 2001 [61], Plocik et al. 2004 [40], and Radwan et al. 2008 [42] showed 70 to 100% identity to 143, 68 and 100 NBS domain sequences identified in this study, respectively (Supplementary File S4).

Following the classification of NBS genes by Shao et al. 2016 [13] and Yu et al. 2014 [5], we classified NBS genes of sunflower into CNL, TNL, RNL and NL groups and their subgroups. We identified 100 genes belonging to the CNL group, with 64 possessing RX_CC like domain, 77 to the TNL group, 13 to the RNL group, and 162 to the NL group. In sunflower, the number of CNLs was found to be higher than that of TNLs, and the ratio of CNLs to TNLs was 1.3:1. The CNL:TNL ratio in the current study is not consistent with the findings observed in some other dicot species such as *A. thaliana* (1:2), *A. lyrata* (1:2), *B. rapa* (1:2), *Eucalyptus grandis* (1:1.25), and *Thellungiella salsuginea* (1:1.5) as numbers of TNLs were higher than CNLs in these species [14,30,65,66,67]. However, grapevine, chickpea, and potato genomes constituted CNL:TNL in a ratio of 4:1 [20,28,57]. The higher number of CNLs in sunflower might suggest the higher contribution of these genes providing resistance against pest or pathogen attack, which warrants future investigation. Furthermore, these groups are classified into subgroups as CNLs were classified into four subgroups [CNL (90), CN (5), CNN (1), CNNL (4)], TNLs into six subgroups [TNL (52), TN (21), TTNL (1), TNLTNL (1), CTNL (1), CTN (1)], RNLs into three subgroups [RNL (10), RN (1), RCNL (2)], and NLs into four subgroups [N (29), NL (125), NN (2), NNL (6)]. The classification is based on the presence of the CC domain named as ‘C’, the presence of TIR domain as ‘T’, the presence of RPW8 domain as ‘R’, the presence of the NBS domain as ‘N’, the presence of two NBS domains as ‘NN’, and the presence of LxxLxxLxx signatures as ‘L’ in the amino acid sequences of the proteins. The CNL type constituted approximately 92% of the genes belonging to CNL subgroup, 67% of the genes belonging to TNL subgroup in the TNL type, 76% of the genes belonging to RNL subgroup in RNL type and 77% of the NL types genes are comprised of NL subgroup genes. The subgroups CN, CNNL, N, NN, and TTNL were also observed in *M. truncatula*, *A. thaliana*, and *B. rapa* [5,19,27]. HanXRQChr03g0067681 and HanXRQChr03g0073241 constituted both RPW8 and coiled-coil domains in the N-terminal and named RCNL, which were also reported in *A. thaliana* and *B. rapa* [5]. HanXRQChr05g0136351 and HanXRQChr06g0184071 possessed both TIR and coiled coil domain in the N-terminal of NBS proteins of sunflower and named CTN and CTNL, respectively. Such subgroups have been previously reported in many legumes and blueberries [16,19].

NBS-encoding genes also called NBS-LRR genes encode proteins having TIR/CC at the N-terminal, NBS domain in the center and LRR at the C-terminal [14]. Among the identified NBS groups, genes belonging to NLs possessed less conserved NBS domain, as only 32% of the genes possessed all three signature motifs, while 57% of the CNLs, 89% of TNLs, and 100% of RNLs possessed all three signature motifs. Of the 100 CNLs, 64 genes possessed Rx_CC like domain in their N-terminal region. The disease resistance protein Rx possess CC domain in the N-terminal, and is expressed against potato virus X in *S. tuberosum* [68]. All TIR1, TIR2, TIR3 and TIR4 were detected in the TNLs of sunflower, which shows the consistency of TIR domain as described in other plant species such as *A. thaliana*, *P. vulgaris*, *G. max*, and *P. trichocarpa* [14,24,63,69]. The characteristic ‘DDVW’ sequence was conserved in kinase-2 motifs of RNL and CNL genes, whereas ‘DDVD’ sequence was frequently found in TNL genes. The ‘TSR’ sequence was highly conserved in RNBS B motifs of the RNLs, while it slightly varies as ‘TTR’ and ‘TTRD’ in the CNLs and TNLs, respectively. This was found to be consistent with the large scale study of NBS proteins in angiosperms [13]. All of the identified NBS proteins possessed MHDL motifs, except for the RNL genes, frequently possessing QHDL motif. Such QHDL motifs were observed in NBS proteins of *P. trichocarpa* [69]. A unique Kelch motif sequence was observed in HanXRQChr02g0052061 protein. Previously, Kelch motifs were reported in the NBS proteins of *B. rapa* [5]. Kelch motif sequences are considered to be signature motif for positive selection mostly found at the C-terminal of F-Box proteins and are well studied in plant species such as *A. thaliana*, *P. trichocarpa*, and *O. sativa* [70].

We further compared our pipeline with another pipeline, RGAugury [35], for the identification of NBS-encoding genes. RGAugury is the integrative pipeline that facilitates the prediction of NBS-encoding genes, RLKs, and RLPs [35]. RGAugury predicted all 352 NBS proteins identified in this study plus five more proteins [HanXRQChr02g0037021 (TN), HanXRQChr09g0240471 (TN), HanXRQChr11g0340171 (CNL), HanXRQChr13g0394521 (TN), and HanXRQChr16g0515381 (CN)] and 25 belonging TX (absence of NBS domain) subclass. These missed proteins were manually checked and NBS domain (PF00931) in HanXRQChr09g0240471, HanXRQChr11g0340171, HanXRQChr13g0394521, and HanXRQChr16g0515381 were absent except in HanXRQChr02g0037021 (could belong to TN subgroup). In addition, we suggest HanXRQChr09g0240471 to be classified as a TX subclass. We found some discrepancies in the CNL group counts between two pipelines. The use of a MARCOIL tool in our pipeline helped with filtering false positives from the CNL group counts, and we could not observe any discrepancies in the TNL group counts between the two pipelines. Furthermore, the RGAugury pipeline could not identify an RNL group of genes that were identified in this study and majorly categorized them to NL group (N and NL subclasses) of genes. The study and identification of TX proteins were beyond the scope of our study as these proteins were filtered out because of the absence of NBS domains. The differences and discrepancies between identification and classification of predicted NBS-encoding genes using our and RGAugury pipelines are represented in Supplementary File S5. In addition, RGAugury was employed to predict proteins belonging to RLP, RLK and Transmembrane-coiled-coil (TM-CC) proteins. A total of 257 RLPs [255-LRR type, 2-lysin motif (LysM) type], 1086 RLKs (368-LRR type, 12-LysM type and 706 Other-receptor type) and 173 TM-CC proteins were predicted in the sunflower (Supplementary File S5). Both RLKs and RLPs play important role in plant development and defense mechanism [4,71]. RLKs such as FLAGELLIN SENSITIVE 2 (FLS2) [72], elongation factor Tu receptor (EFR) [73], systemin cell-surface receptor (SR160) [74], Xa21 [75], ERECTA RLK [76] and many more are well characterized that are mainly involved in detection of pathogen associated molecular patterns (PAMPs). On the other hand, RLP (lacking Kinase-2 domain) such as *Arabidopsis* CLAVATA2 (CLV2, AtRLP10) [77] is involved in the development of meristem and Cf is involved in pathogenesis against *Cladosporium fulvum* in tomato [78].

### 4.2. Gene Location, Clustering, K_a_/K_s_ Values and Structural Variation

A variety of clustering patterns of NBS-encoding genes, frequently observed in almost all plant species, is one of the major reasons for rapid evolution of the NBS genes [14,79]. The NBS genes of sunflower formed 75 clusters, 25 of which reside in chromosome Ha13, 73 out of 200 (~37%) genes. In *M. esculenta*, 143 NBS genes positioned in 39 clusters [64]. In *C. sativus*, 33 *NBS* genes were located in nine clusters [33]. The average number of NBS proteins per cluster in sunflower was approximately 2.7, lesser than ratios in Solanaceae species such as tomato (3.48), potato (4.65), pepper (3.44) [80], Brassicaceae species such as *B. oleracea* (3.04), *B. rapa* (2.7), *A. thaliana* (2.8) [5], Fabaceae species such as *G. max* (4), *V. vinifera* (6), *M. truncatula* (5) [16], *Gossypium* species such as *G. arboretum* (3.4), *G. raimondii* (5.5), *G. hirsutum* (5.3), and *G. barbadense* (3.5) [34]. Both segmental and tandem duplications are responsible for the formation of new clusters that generate intraspecific variation by processes such as unequal crossing over [9,14,81]. However, NBS-encoding genes do not undergo high rates of mutation and maintain both intra- and inter-specific variation [9]. The average exon counts of sunflower CNLs (2.7 exons per gene) and TNLs (6.1 exons per gene) were consistent with CNLs (2.7 exons per gene) and TNLs (5.1) of *Arabidopsis* [14]. This implies a high number of exons of TNLs and RNLs could help with generating diverse resistance proteins through alternative splicing. All NBS types showed *K_a_*/*K_s_* values of less than one, indicating that these genes are under the influence of purifying selection.

### 4.3. Phylogenetic Relationships, Homology, Synteny and Expression Analysis

Sunflower CNL genes were similar to *C. sativus* CNL genes while compared to their respective TNL genes [33]. However, the CNL clade size in sunflower is different from *Arabidopsis*, as TNL clades constitute larger numbers of genes than CNL clade [14]. Subclades CC (I) possessed gene members with introns in range of one to ten, and CC (II) constituted gene members with introns in the range of zero to one. Other subclades, CC (III) and CC (IV) constituted gene members with introns in the range of zero to two and CC (V) and CC (VI) constituted gene members with introns in the range of zero to four. Only *HanXRQChr02g0057361*, *HanXRQChr02g0057351*, and *HanXRQChr13g0425771* in the subclade CC (VI) possessed in the range of five to seven. Similarly, subclade TIR (II) possessed gene members with introns in the lowest range (three to five). TIR (I), TIR (III), TIR (IV), TIR (V) and TIR (VI) gene members possessed introns in range of 3 to 17, 2 to 7, 1 to six, 1 to 15, and 1 to 13, respectively. Similar patterns were also observed in the phylogenetic tree of CNL and TNL in *C. sativus* [33]. The differences in the clade pattern with correlation to introns in two gene families suggest the role of intron loss and gain in the structural evolution of the NBS genes as suggested by Wan et al. 2013 [33]. In addition, the position, presence or absence, and phase of introns often play important roles in phylogeny [82].

We found that RNLs were nested within the clade of TNLs in sunflower (a member of the Asterids lineage) although RNLs in the families Brassicaceae and Fabaceae (Rosids lineage) were found to be related to CNLs [15,30]. The lineage of Asterids is believed to have evolved from the rest of Angiosperms (Rosids + monocots + basal Angiosperms) little over 100 million years ago (MYA) [83]. A large-scale study of Angiosperms NBS genes also concluded that RNLs were sister to the CNLs [13]. However, these earlier studies did not include *H. annuus* in the analysis as the genome was not available by then. Our results indicate a surprising position of RNLs within TNLs in sunflower making the clades of TNL and CNL potentially paraphyletic. Upon reconstruction of the phylogenetic tree with *Arabidopsis* NBS genes, RNL genes of sunflower were observed in a CNL-A clade (although it is consistent with the previous study) [14]. The CNL-A clade did not consist of any sunflower CNL gene members besides RNLs. Further study on comparative genomics or transcriptomes across the Asterids lineage can confirm whether CNL genes are completely absent in the lineage. Shao et al. 2016 [13] suggested that RNLs were derived from *ADR1* and *NRG1*, and two ancient lineages separated before the Angiosperms diversified. The RNL genes, *ADR1* and *NRG1*, have been characterized in *Arabidopsis* and *Nicotiana*, respectively. A separate tree, constructed to observe the relationships among sunflower RNLs and *Arabidopsis* RNLs, formed two clades. The sunflower RNL genes *HanXRQChr02g0046611* and *HanXRQChr05g0129181* were nested with *AT4G3330* (*ADR1-L1*), *AT1G33560* (*ADR1*) and *AT5G04720* (*ADR1-L2* or *PHX21*), with bootstrap support of 90%. On the other hand, *HanXRQChr02g0048181*, *HanXRQChr11g0331571*, *HanXRQChr03g0067681*, *HanXRQChr0073241*, and *HanXRQChr04g0095241* were nested with *AT5G66630* (RNL) and *AT5G66910* (homologous to *NRG1*), with bootstrap support of 63%. This suggests that the sunflower RNLs mentioned above are orthologous to the ADR1 and NRG1 homologs of *Arabidopsis*. ADR1 proteins play a role as helper genes for receiving signals from the R genes in downstream signaling of effector-triggered immunity [84]. Similarly, NRG1 proteins help the N protein during the pathogenesis by the tobacco mosaic virus [85]. Since they are not directly involved in detecting the pathogen effectors, they are not much influenced by a selection pressure due to the pathogens [13]. Only 5.8% of the total NBS genes in sunflower are RNL genes which is consistent with other species, such as *A. lyrata* (2.5%), *A. thaliana* (4.2%), *B. rapa* (4.4%), *Capsella rubella* (4.7%) and *T. salsuginea* (5.7%) [30]. Other results from this study that separate RNLs from the rest of the NBS genes include their highest average number of exons per gene and lowest average *K_a_/K_s_* ratios values for the clade. This supports the hypothesis of high conservation and slow evolutionary rates among the RNL genes [86].

Sunflower NBS proteins identified in this study formed clades with reference proteins such as Pi36, Pl8, Rps2, VAT, RPG1, Gro1.4, RY-1, and N proteins, suggesting their homologous relationships (Figure S13). The sunflower TNL proteins are inferred to be orthologous to *S. tuberosum* nematode resistance protein (Gro1.4) [87], *S. tuberosum* subsp. *andigena* RY-1 (conferring resistance to potato virus Y) [88], and *N. glutinosa* Tobacco Mosaic Virus resistance (N) gene [89]. Similarly, sunflower CNL proteins are inferred to be orthologous to *A. thaliana* RPS2 (Resistant to *P. syringae* 2) [90], *Cucumis melo* VAT (resistance to *Aphis gossypii*) [91], *H. annuus* Pl8 [43], *O. sativa* Pi36 (conferring resistance to Blast fungus) [92], and *H. vulgare* subsp. *vulgare* RPG1 (conferring resistance to stem rust fungus) [93]. The BLAST investigation of sunflower NBS proteins with reference proteins available on http://www.prgdb.org has shown some of them to be the possible homologs of the reference proteins (Table S7). Sunflower NBS proteins such as HanXRQChr13g0425411, HanXRQChr13g0425361, and HanXRQChr13g0425431 showed greater than 80% sequence identity to the *H. annuus* gene, *Pl8* gene (CNL). The *Pl8* gene is involved in conferring resistance to *P. halstedii*, a causative agent to downy mildew [43]. HanXRQChr04g0123041, belonging to the NL group has shown homology to *L. esculentum* EIX receptor 2 (*Eix2*), a gene that encodes receptor-like proteins (RLPs) involved in detecting ethylene-inducing xylanase, a fungus elicitor [94]. Other inferred homologs include HanXRQChr17g0552491 to MLA10, HanXRQChr13g0420141 to N, HanXRQChr17g0552491 to both MLA12 as well as MLA13, and HanXRQChr17g0552491 to Sr33. The *MLA* locus is highly polymorphic and encode allelic CNL type resistance proteins such as MLA1, MLA2, and MLA3 that confer resistance to barley powdery mildew fungus (*Blumeria graminis* f. sp. *Hordei*, *Bgh*) [95]. Another protein, Sr33, which belongs to the CNL type, confers resistance to a wheat stem rust pathogen, *Puccinia graminis f*. sp. *tritici* [96]. We were able to access expression profiles for only a few unique sunflower NBS proteins because of the duplicated names found for corresponding *H. annuus* r1.2 annotations compared to *H. annuus* r1 annotations. From the available expression data, it can be deduced that NBS genes can be expressed at a basal level with tissue specificity in unchallenged conditions [97]. In the expression dataset, most of the NBS genes were found to have a minimal to no expression value possibly as a result of low sequencing coverage, or their expression dependent on infection of pathogens or due to a pseudogenization, which was also noted by Frazier et al. 2016 [98]. Thus, detailed transcriptomic and proteomics studies are warranted to functionally characterize the sunflower NBS genes, particularly challenging the plant by various pests and pathogens through carefully crafted experimental designs.

## 5. Conclusions

We identified 352 NBS genes in sunflower and studied their clustering, phylogenetic relationships, gene homology and functional divergence. These genes formed clusters and showed structural conservation in signature domains and exon/intron architecture in CNL, TNL and RNL types of NBS genes. The RNLs belonged to the CNL-A clade, which in turn was found nested within the TNL clade, making both CNL and TNL clades paraphyletic. This warrants further rigorous analysis. All of the NBS-encoding genes have undergone purifying selection and available expression data have revealed their functional divergence. We confirmed homology of sunflower NBS genes to multiple previously characterized *Pl8*, *LeEIX1*, *MLA10-13*, *Sr33* resistance genes. Further characterization of the NBS genes will help us to understand resistance pathways and to develop durable resistance necessary for crop improvement in sunflower, one of the major oilseed crops in the world.

## Figures and Tables

**Table 1:** Nucleotide Binding Site (NBS)-encoding proteins in sunflower in relation to 15 other plant species.

Protein Letter Code	Number of Proteins															
	Ha *	**At ^a^**	**Gm ^b,c^**	**Mt ^a^**	**Bo ^a^**	**Br ^a^**	**Tc ^a^**	**Pt ^a^**	**Vv ^a^**	**Ca ^d^**	**Cs ^e^**	**Pv ^f,c^**	**Lj ^f^**	**Cc ^f^**	**Gs ^f^**	**Ga ^g^**
CNL	90	17	95	152	6	19	82	120	203	19	17	31	11	37	47	80
CN	5	8	-	25	5	15	46	14	26	33	1	40	26	41	62	44
CNNL	4	-	5	-	-	2	-	-	-	1	-	-	-	-	-	-
CNN	1	-	-	-	-	-	-	-	-	-	-	-	-	-	-	-
RNL	10	2	6	-	1	4	-	-	-	2	2	-	-	-	-	3
RN	1	3	-	-	2	1	-	-	-	2	-	-	-	-	-	-
RCNL	2	-	-	-	-	-	-	-	-	-	-	-	-	-	-	-
TNL	52	79	126	118	40	93	8	78	97	6	11	81	16	47	49	5
TN	21	17	22	38	29	23	4	10	14	7	2	11	53	36	76	2
TNNL	0	1	-	-	1	4	-	-	-	-	-	-	-	-	-	-
TTNL	1	-	-	-	-	-	-	-	-	1	-	-	-	-	-	-
TNLTNL	1	-	-	-	-	-	-	-	-	-	-	-	-	-	-	-
CTNL	1	-	-	-	-	-	-	-	-	-	-	-	-	-	-	-
CTN	1	-	-	-	-	-	-	-	-	-	-	-	-	-	-	-
N	29	26	4	328	53	29	53	62	36	14	1	59	82	136	213	59
NL	125	20	73	-	24	27	104	132	159	12	23	20	18	56	58	53
NN	2	-	-	-	3	2	-	-	-	1	-	-	-	-	-	-
NNL	6	-	-	-	-	3	-	-	-	-	-	-	-	-	-	-

Note: Ha: *Helianthus annuus*; At: *Arabidopsis thaliana*; Gm: *Glycine max*; Mt: *Medicago truncatula*; Bo: *Brassica oleracea*; Br: *Brassica rapa*; Tc: *Theobroma cacao*; Pt: *Populus trichocarpa*; Vv: *Vitis vinifera*; Ca: *Cicer arietinum*; Cs: *Cucumus sativus*; Pv: *Phaseolus vulgaris*; Lj: *Lotus japonicas*; Cc: *Cajanus cajan*; Gs: *Glycine soja*; Ga: *Gossypium arboretum* (* = this study, ^a^ = [5], ^b^ = [23], ^c^ = [24], ^d^ = [20], ^e^ = [33], ^f^ = [16], ^g^ = [34]).

## Supplementary Materials

The following are available online at http://www.mdpi.com/2073-4425/9/8/384/s1. Figure S1: Predicted protein domains in sunflower CNL protein sequences with number of LxxLxxLxx signatures in parentheses. Figure S2: Predicted protein domains in sunflower TNL protein sequences with the number of LxxLxxLxx signatures. Figure S3: Predicted protein domains in sunflower RNL protein sequences with number of LxxLxxLxx signatures. Figure S4: Predicted protein domains in sunflower NL protein sequences with the number of LxxLxxLxx signatures. Figure S5: Conserved domains of sunflower CNL genes predicted by MEME analysis. Figure S6: Conserved domains of sunflower TNL genes predicted by MEME analysis. Figure S7: Conserved domains of sunflower RNL genes predicted by MEME analysis. Figure S8: Chromosomal distribution of NBS genes in a sunflower (*n* = 17). Figure S9: Exon–intron architecture of the coding sequences of CNL genes in sunflower. Figure S10: Exon–intron architecture of the coding sequences of TNL genes in sunflower. Figure S11: Exon–intron architecture of the coding sequences of RNL genes in sunflower. Figure S12: Exon–intron architecture of the coding sequences of NL genes in sunflower. Figure S13: Maximum likelihood (ML) tree of the NBS amino acid sequences of the CNL, TNL and RNL genes from sunflower along with those of previously characterized CNL, TNL and RPW8 type genes. Figure S14: Syntenic relationships between chromosomes of *Arabidopsis* and sunflower. Figure S15: Identified genes in different clusters showing differential expression on all tissues in sunflower. Table S1: List of NBS gene accessions, their type, number of LxxLxxLxx signatures, exon/introns number, protein sequence length, gene orientation, amino acid length and amino acid sequences. Table S2: List of *NBS* genes of sunflower with nuclear localization signal (NLS) peptides. Table S3. Conserved MEME motifs in sunflower CNL family of proteins. Table S4. Conserved MEME motifs in sunflower TNL family of proteins. Table S5. Conserved MEME motifs in sunflower RPW8 family of proteins. Table S6: List of genes with their clusters and chromosomal location. Table S7: BLAST result of NBS genes against reference genes of Plant Resistance Genes database (PRGdb; http://prgdb.org) with a cutoff E-value of 0.01. Supplementary File S1: The motif sequence logos in the sunflower CNL family of R proteins. Supplementary File S2: Sequence alignment of the NBS domains belonging to different groups in fasta format. Supplementary File S3: Newick files for phylogenetic trees shown in Figure 3, Figure 4 and Figure 5. Supplementary File S4: BLASTP result of NBS domains of the genes identified in this study and those previously identified by Gedil et al. 2001 [61], Plocik et al. 2004 [40], and Radwan et al. 2008 [42]. Supplementary File S5: Identification and classification of NBS-encoding genes using current pipeline (this study) compared to those predicted by RGAugury pipeline, and a list of RLPs, RLKs and TM-CC proteins in sunflower.
