# Supplementary material for: Genome-Wide Identification of NBS-Encoding Resistance Genes in Sunflower (Helianthus annuus L.)
Source: Genes (Basel). 2018 Jul 30;9(8):384. doi: 10.3390/genes9080384 (PMC6115920; doi:10.3390/genes9080384)
Supplement: Supplementary file 1 [file genes-09-00384-s001.zip › Supplementary Files/Table S3-S5.pdf]

Table S3. Conserved MEME motifs in sunflower CNL family of proteins. The motif logos represented in column three are shown in Supplementary File S1a.

Domain	Motif number	Motif	Motif Sequence	Length	E value
**CC**	**Motif 14**	**C1**	MA[ED][TA][LAI]A[SN][EA]L[LV]KV[LV][FV][KE]K[LM]T[DS]EAFKR[IF]AR[AS]Q	29	7.5e-1109
	**Motif 3**	**C2**	[TD][LF]S[RQ]IQ[DA][LV]LNDA[SE][QE]KE[VI][TK][HE][KE][SA]VK[LSE]WLNAL[QR][HS][LA][AS]YD[IV][DE][DN]VLDD[LVI][AS]TE[AD][ML][HL]R	50	1.0e-2772
	**Motif 10**	**C3**	P[ST]CCTNFSL[TS]HR[LM][SH][PH]KLDSI[NT][RT][EK]L[EQ][ND]LEK[RQE]KTDLGL	36	3.0e-1407
**NBS**	**Motif 2**	**P loop**	[VI][LV]PI[VW]GMGG[VL]GKTTLARL[LV]YN	21	8.7e-1518
	**Motif 8**	**RNBS A**	[HY]FE[LP]KAWV[CY]VSDDF[DQ][IV]FKI[ST][KD][TA]I[LFI]Q	25	3.8e-1315
	**Motif 4**	**Kinase 2+RNBS B**	KRFLLV[LV]DDVWSENY[GD]DWE[NT]LVRPF[LH]SCAPGS[RK]IIMTTRKE	41	4.2e-2089
	**Motif 15**	**RNBS C**	LS[HNE]ED[AS][LW][RSL]LF[AK][LVK]HA[LF]	15	4.0e-599
	**Motif 7**	**GLPL**	PIG[ER]GIVKKCGGLPLA[LI]K[TVA][IL][GA]R[LS][LM]R	25	6.7e-183
	**Motif 5**	**RNBSD+CNBS1**	YH[DE]LS[AP][DH]LK[QR][LC]FAY[CF]SLFPK[DG][FY]LFDK[ED]EL[VI][LS]LW[MV]AEGF[LI]NP	41	3.1e-2273
	**Motif 1**	**MHDL**	RSFFQHAPNDESLF[IV]MHDLMNDLAT[FS]VAGEFFLRFDN[HE]M[KE]T	41	3.7e-1966
**LRR**	**Motif 18**	**L1**	[FCY]YLS[SN]KIL[DV]DLLPEL[TP]LLRVLS	22	3.3e-761
	**Motif 6**	**L2**	PE[FS]IG[ST]LKHLRYLNLSRTNI[KE]ELPE[NS][VI]GNLYNLQTLI[VL][SF]GC	41	1.1e-2102
	**Motif 16**	**L3**	[GS]LTKLP[KN][SG][FL][LS][KN]LK[NRK]LRH[LF]DIR[DG]T	23	6.9e-735
	**Motif 9**	**L4**	GFAI[NT]ELK[GD]LTNLHG[EK][VI]SIEGLHKVQSA[KM]HAREANL	36	2.8e-1446
	**Motif 17**	**L5**	CL[KR]ELV[IV][KR][ND]CP[NQ]L[IV][DQN]VS[LP][EQ]ALPSL[NKR]VLEI[DYS][RKE]C[GD][SD]GVLRSLVQ[VA]AS[SG]VTKL	50	6.9e-801
	**Motif 19**	**L6**	[KE]I[ES]SISGL[TDN][DY][VE]VWRGVIEYL[GK][AE]VE[EY]L[SR]IKNCNEI[RK]YLWES[EM][AT][ES][AK][SA][KL][VIL]L[VL][RN]	50	5.2e-625

Table S4. Conserved MEME motifs in sunflower TNL family of proteins. The motif logos represented in column three are shown in Supplementary File S1b.

Domain	Motif number	Motif	Motif Sequence	Length	E value
**TIR**	**Motif 1**	**TIR 1**	FRGEDTRK[TNS]F[VT]DHLY	15	1.7e-836
	**Motif 2**	**TIR 4**	[[IV][IV]V[FL]SKNYASSSWCLDEL[VA][KL]I[ML]E[CQ][QK]	25	6.4e-1078
	**Motif 3**	**TIR2**	[HQ][TI][VA]YP[VI]FY[DH]V[ED]P[ST][DE]VR[KN]Q[SK]GS[VF]GEA	25	1.0e-1170
	**Motif 9**	**TIR 3**	W[RK][KN]AL[KT]E[AV]A[ND]L[SA]GW[EVD]LK[NG]TA[ND]GHEA	25	4.3e-816
**NBS**	**Motif 4**	**P loop**	VR[MI][ILV]GI[KWC]G[MI]GG[GIS]GKTTLA[RK][AY][VI][FY][DN]xI	25	1.6e-1149
	**Motif 20**	**RNBS A**	[GD]L[LK]KLQ[KE][QK][LI]L[SK]D[IVL]L[KNG]	25	2.5e-291
	**Motif 5**	**Kinase 2**	[KR]VL[VIL]VLDD[VI]DDI[DE]QLEALAG[TS][HP]NWF	25	2.3e-1000
	**Motif 10**	**RNBS B**	[KG][PS]GSRIIITTRDE[QH][VL]LIAH[RK]V	21	7.3e-631
	**Motif 11**	**RNBS C**	L[SN]D[ED]E[AS][IL]EL[FL][SN][RK][HY]AF	15	1.8e-486
	**Motif 6**	**GLPL**	VV[HS]Y[AC][AG]GLPL[AT][LI]KVLGS[FS]L[CY][GD]	21	2.3e-843
	**Motif 17**	**TNBS I**	[RK]LKTIP[LNE]KE[TIV]L[KE][KR]L[EK][LI]S[YF]DGL	21	2.4e-378
	**Motif 12**	**RNBS D**	[EQY]KE[LI]FL[DH][IV]AC[FI][FL][KRV]G[EW]	21	7.2e-415
	**Motif 7**	**MHDL**	MHD[LH][LI][QE]EMG[RK]NIVRR[EL][SH]P[DN]EP	15	1.2e-522
**LRR**	**Motif 18**	**L1**	[KR][HR]SR[LV]WIKE[ED][IS][EY]D[VI]L	15	1.6e-376
	**Motif 16**	**L2**	L[RQ][WY]LCWHG[YF]P[LF][SK][CS][LIF]P[KS][TD][FL]Q[AMP][NE]	21	5.6e-472

Table S5. Conserved MEME motifs in sunflower RPW8 family of proteins. The motif logos represented in column three are shown in Supplementary File S1c.

Domain	Motif number	Motif	Motif Sequence	Length	E value
**RPW8**	**Motif 19**	**RPW8-1**	DR[PR][EK]EET[EDTK][MR]F[IM][FIS][HLY]L[EK][QNK][GA][KE][EK]LV[VL]KC[SE]	25	1.5e-055
	**Motif 8**	**RPW8-2**	WN[VL]Y[KQ][KR][FY]V[HY][AS][NS]KL[ID][RK]L[ND][KNH][ESK]LLR[FY][FV]Q	25	3.3e-092
**NBS**	**Motif 1**	**P loop**	V[VL]VVS[AG][PA]GG[CS]GKTTL[VA][KTR][ML][LF]C[HR]D[NPD][ED][IV]	25	4.4e-166
	**Motif 14**	**RNBS A**	[KQ][GE]IFG[ED]NI[LFY][FY]VT[VI]S[RE]T[TPY][SND]LK[VT]	21	2.0e-062
	**Motif 18**	**Kinase 2**	LLVLDDVW[SA]ES	11	3.4e-052
	**Motif 5**	**RNBS B**	IQD[LF][KM]F[KQ][IS]PG[YC]K[IV]LVTSR[FI][LT]F	21	2.2e-110
	**Motif 6**	**RNBS C**	TY[EK]L[GS]LLND[EQ]DA[RT][TS]L[FL]C[HY]SAF[PS][REC]D[GS]	25	1.4e-109
	**Motif 3**	**GLPL**	KCCKG[HLF]PLAL[TSK]V[IV]G[AG]SL[RCK][GD]QP[EV][LA]KW	25	6.6e-141
	**Motif 2**	**RNBS D**	C[FY]LDLG[SL]FPED[QEK][KR]I[ASP]A[TS][AVT]LMDMW[VA][EH]	21	7.2e-415
	**Motif 4**	**QHDL**	VTQHD[LVM][LM]R[ED]LAIH[LM]SSQE[PS]	19	3.8e-104
**LRR**	**Motif 12**	**L1**	LK[VA]L[NIV][IV]T[NS]Y[GS][YI]Y[PF][ST]E[LI][HQ][NE][LF][PS]	20	1.5e-071
	**Motif 17**	**L2**	NL[QR]KLS[LF]IMC[KE]IGNA[FL][EN]SCT	20	3.0e-057
	**Motif 9**	**L3**	C[DY]D[LM][KV][TE][FL]P[STA][SM]LCN[LV][VK]RLKKLSITNC	25	7.1e-101
	**Motif 11**	**L4**	[FL]G[NS]L[SL]NLE[IV]LR[LV][HA]SCT[KR]LK[TK]LPES[IM]	25	8.2e-083
	**Motif 13**	**L5**	PE[HEQ]IGELG[GS]LR[TV][IL][DK]M[RS]GC[TH]GL[EH]ELP	25	8.06-064
