# Supplementary material for: Genome-Wide Identification of NBS-Encoding Resistance Genes in Sunflower (Helianthus annuus L.)
Source: Genes (Basel). 2018 Jul 30;9(8):384. doi: 10.3390/genes9080384 (PMC6115920; doi:10.3390/genes9080384)

Supplementary File S1a. The motif sequence Logos in the sunflower CNL family of R proteins

Motif 14: C1

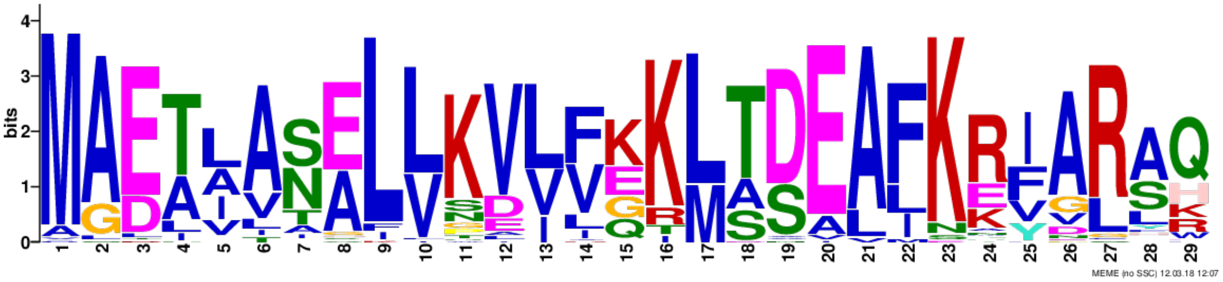

Motif 3: C2

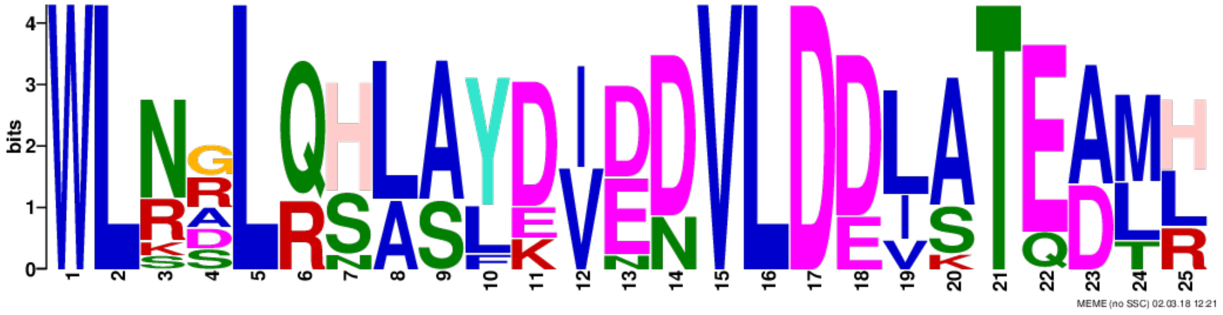

Motif 10: C3

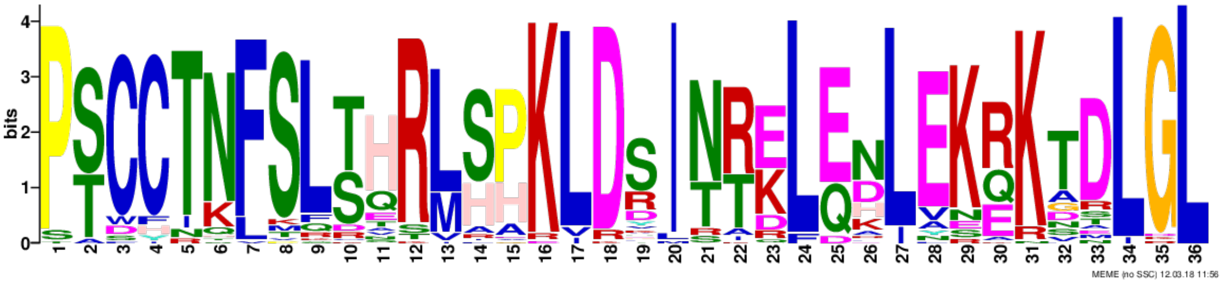

Motif 2: P loop

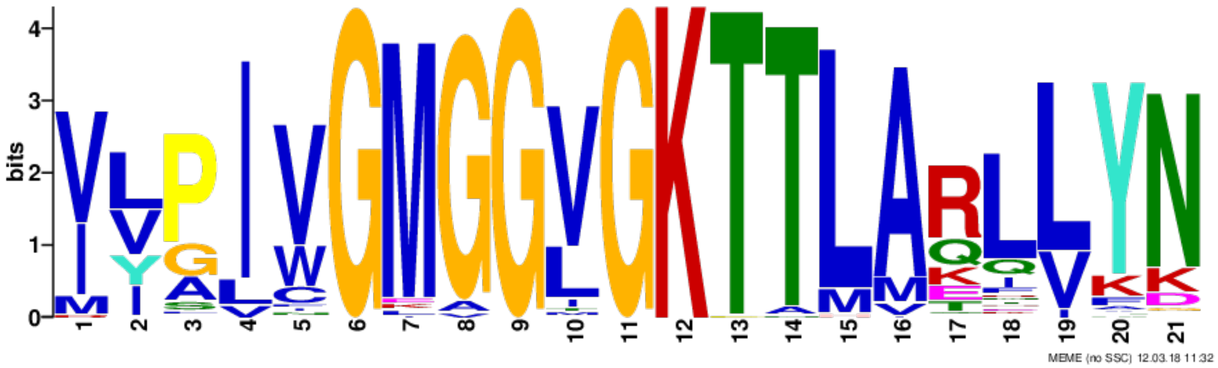

Motif 8: RNBS A non TIR

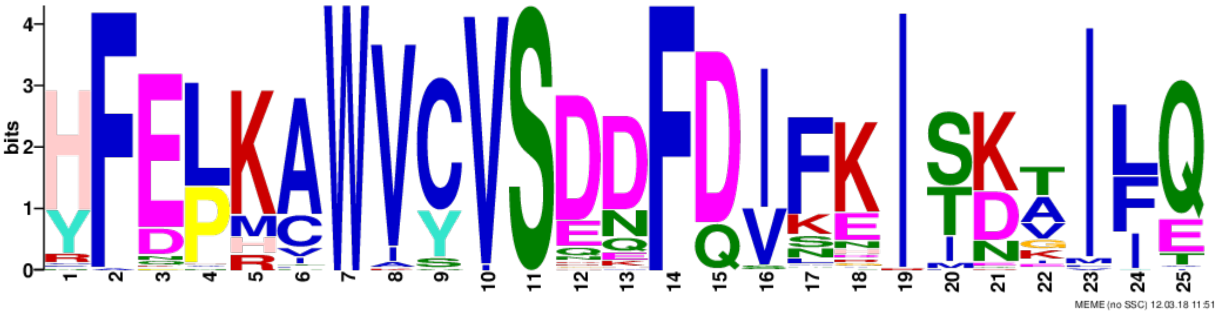

Motif 4: Kinase 2 + RNBS B

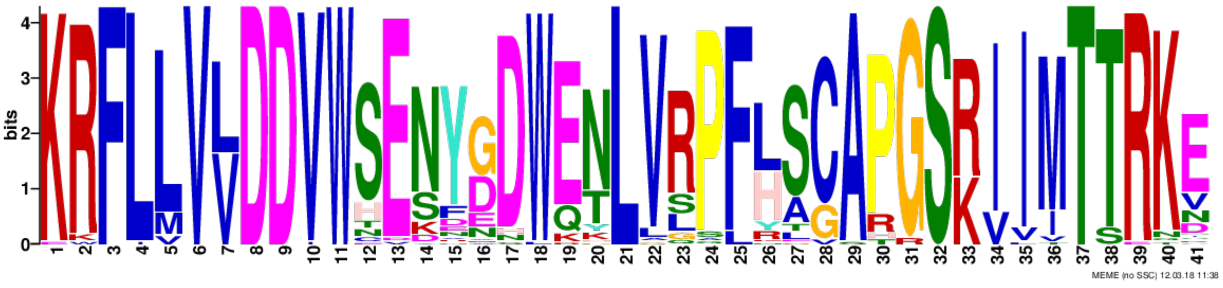

Motif 15: RNBS C

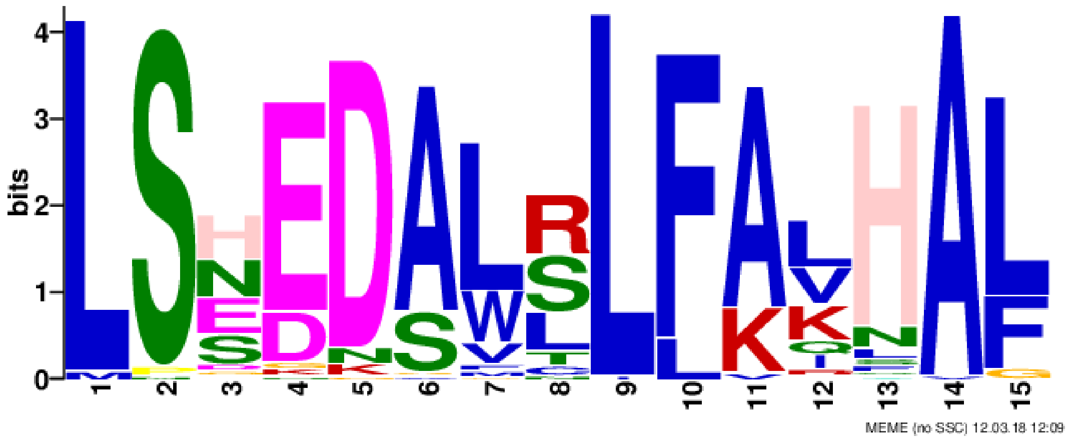

Motif 7: GLPL

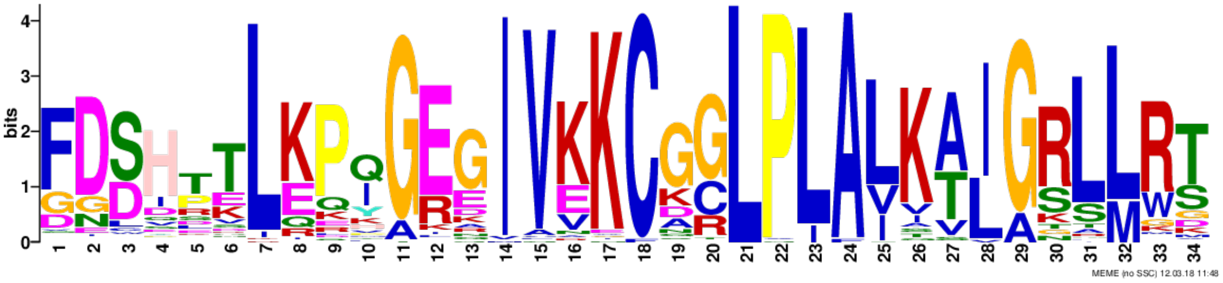

Motif 20:

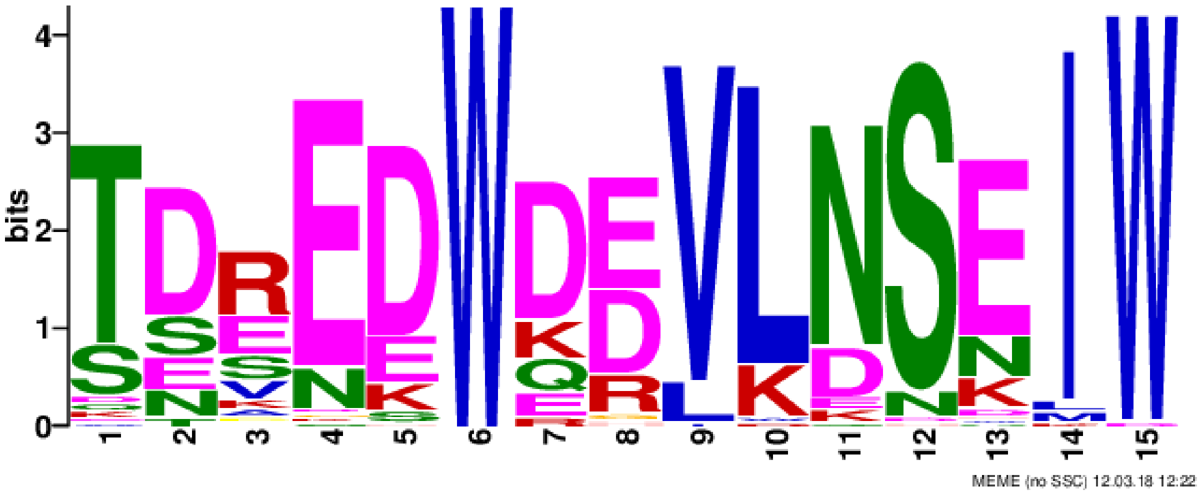

Motif 5: RNBSD + CNBS1

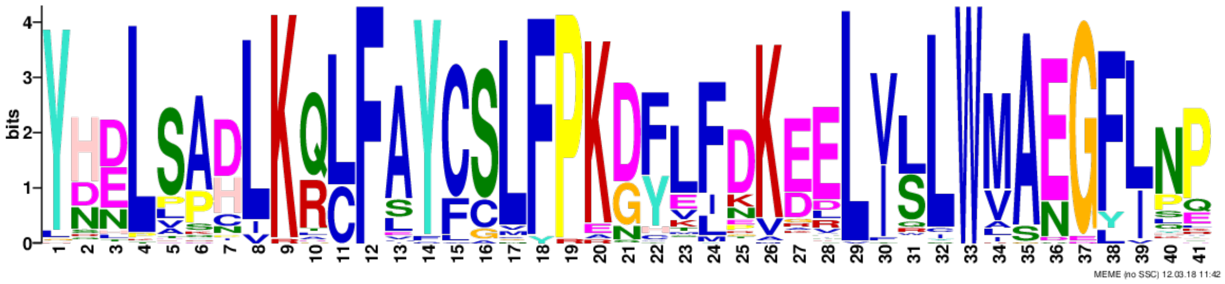

Motif 1: MHDL

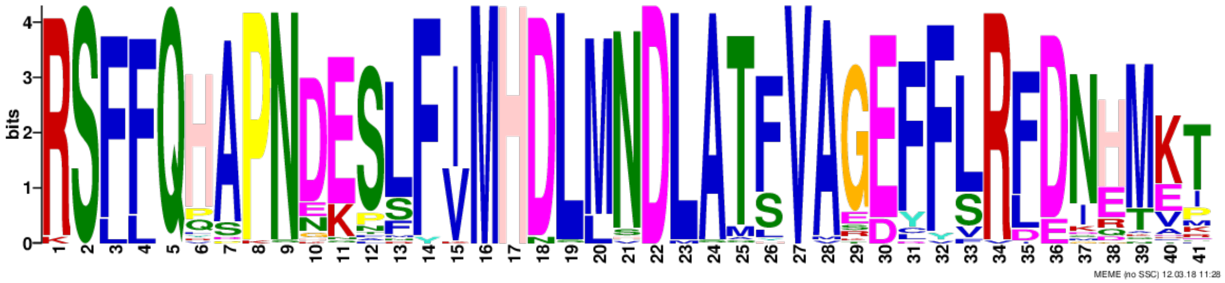

Motif 11:

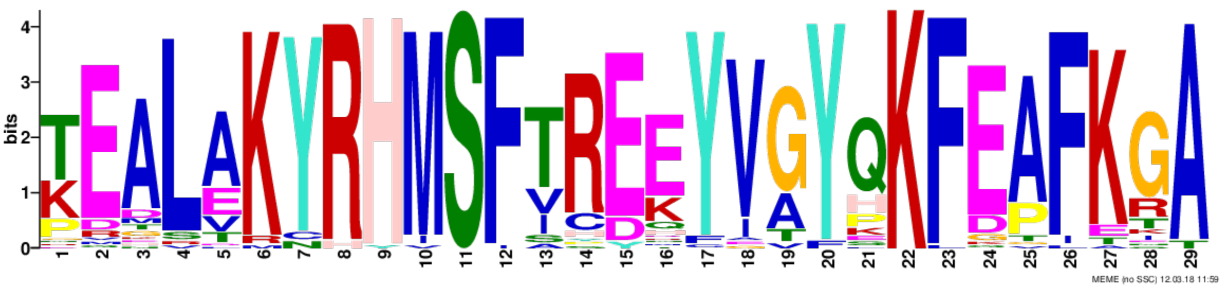

Motif 18: L1

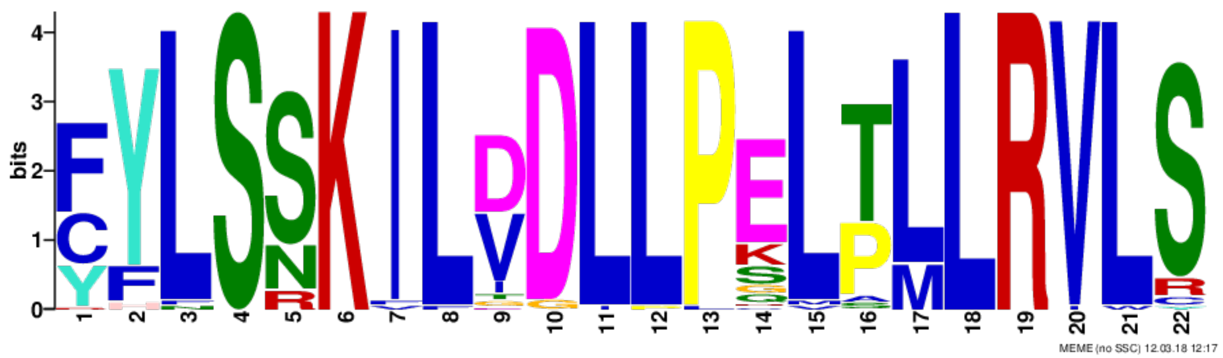

Motif 6: L2

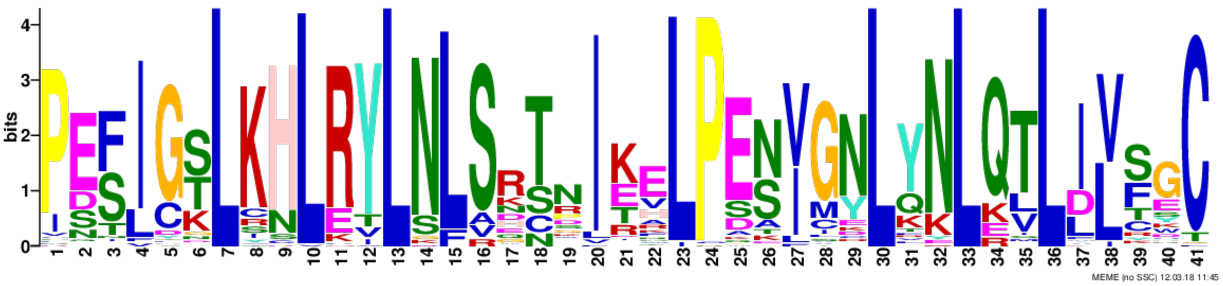

Motif 16: L3

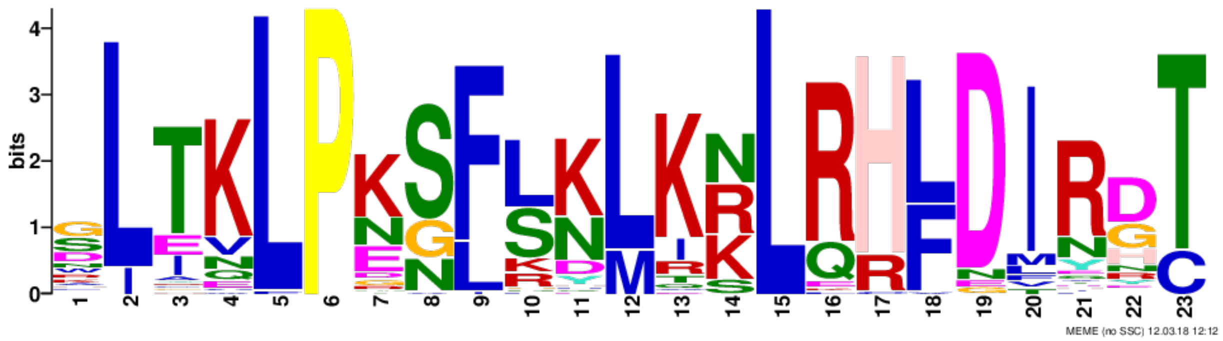

Motif 9:L4

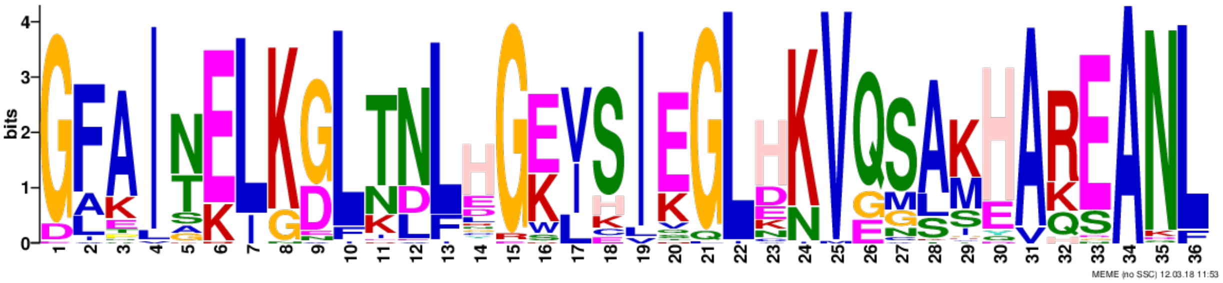

Motif 12:

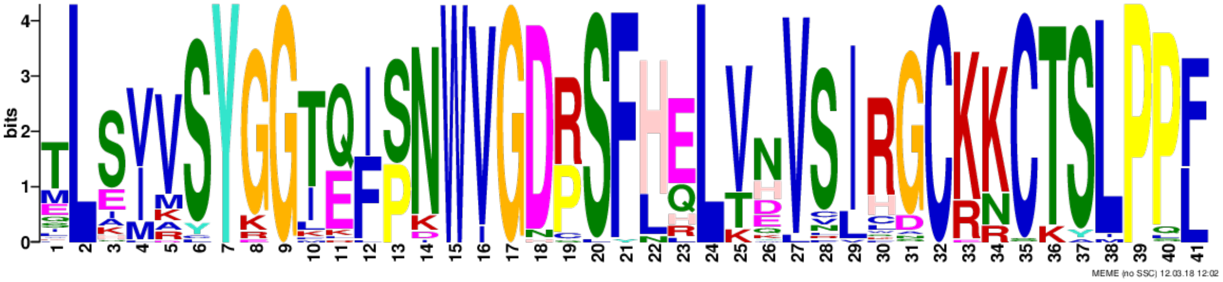

Motif 13:

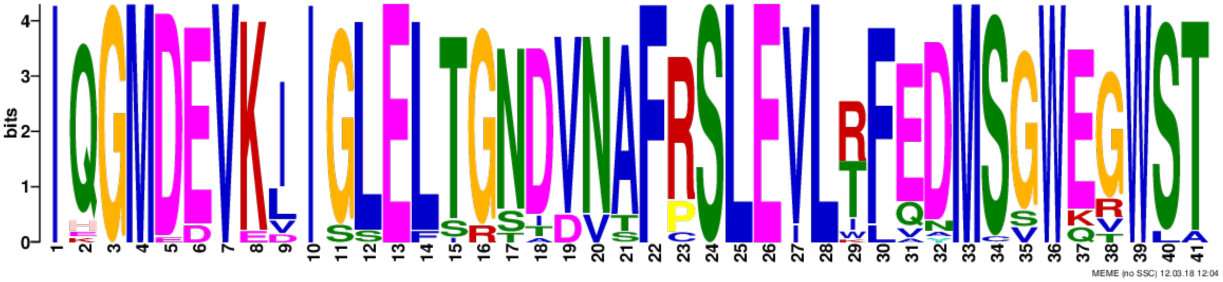

Motif 17:L5

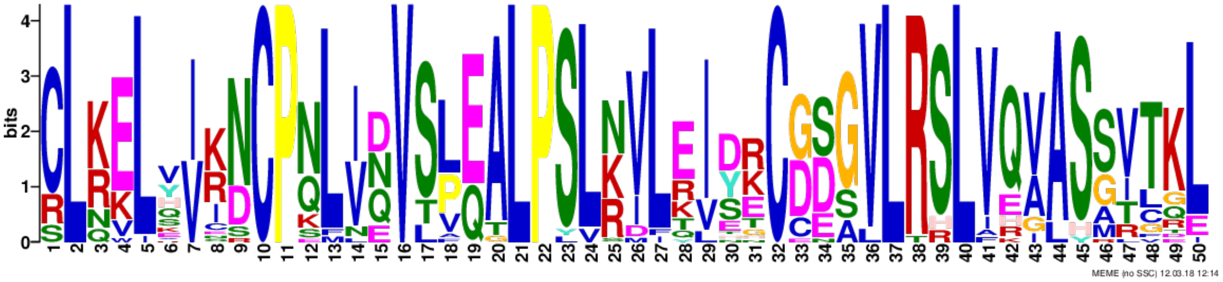

Motif 19:L6

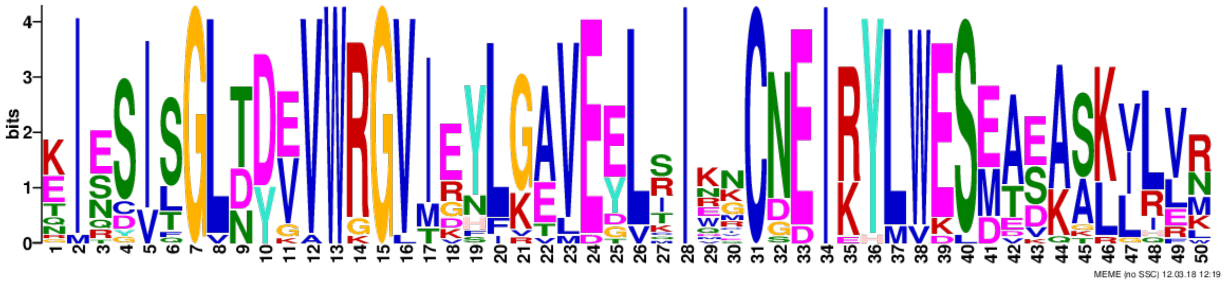

Supplementary File S1b. The motif sequence Logos in the sunflower TNL family of R proteins

Motif 19:

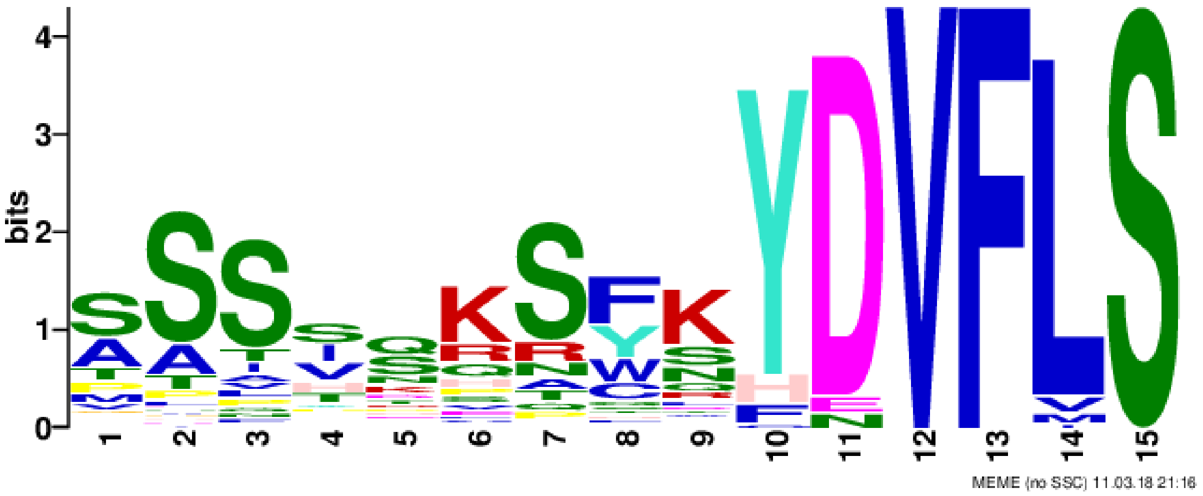

Motif 1: T1

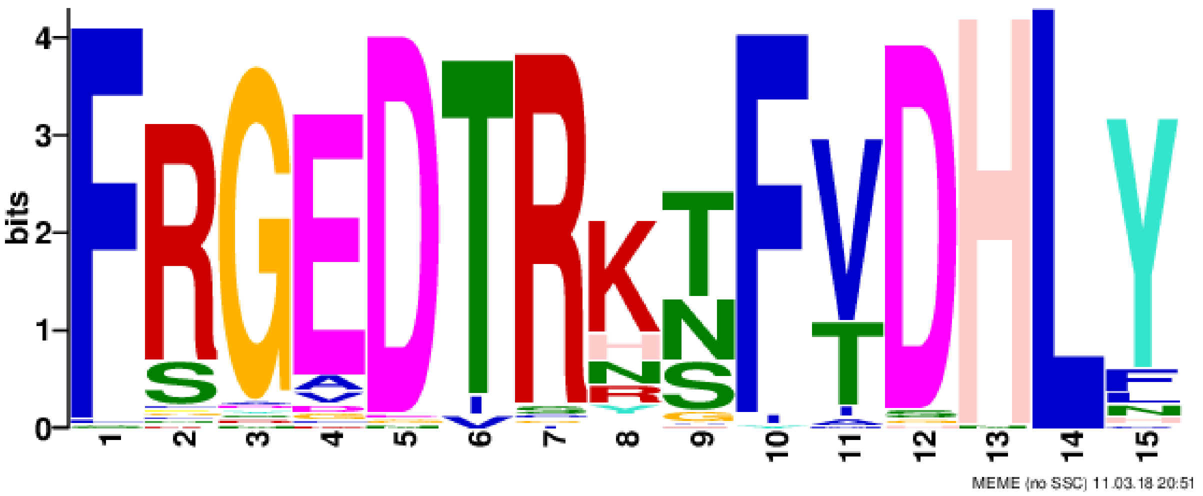

Motif 8:

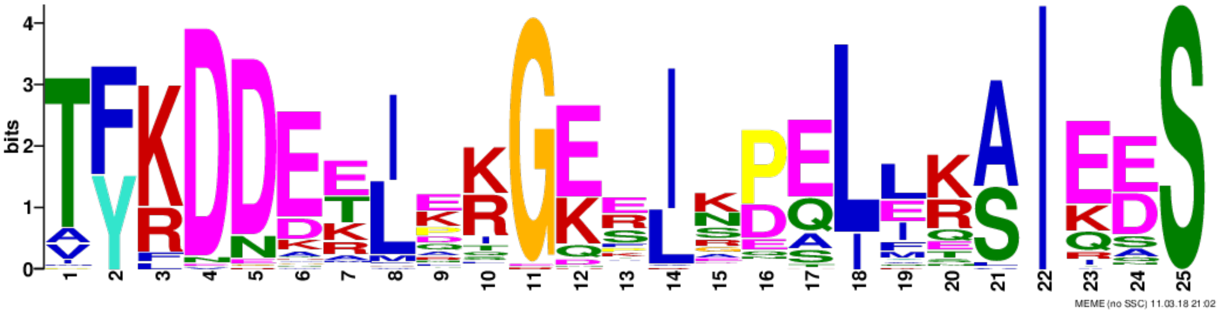

Motif 2: TIR4

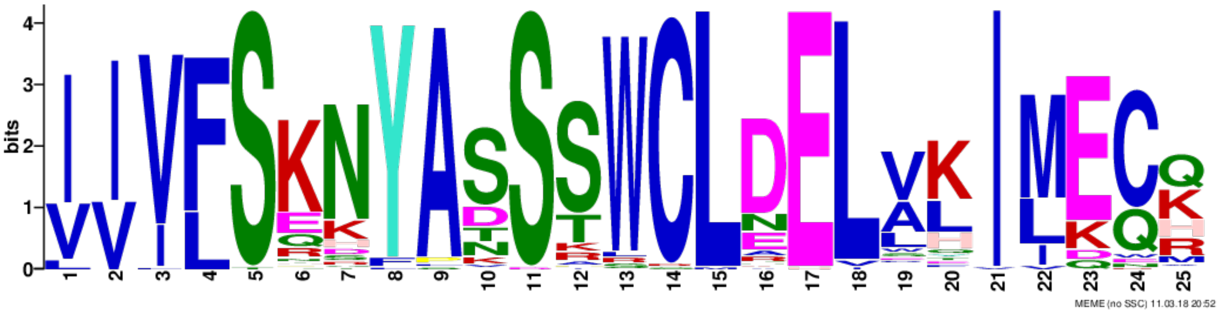

Motif 3: TIR 2

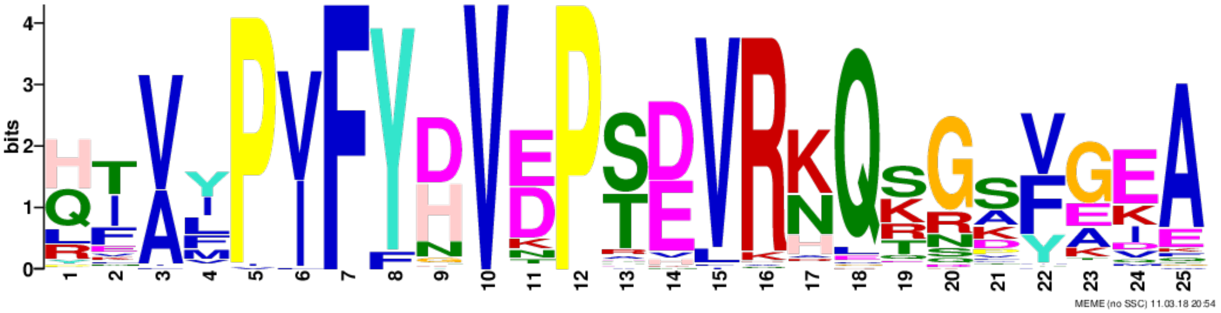

Motif 9: TIR 3

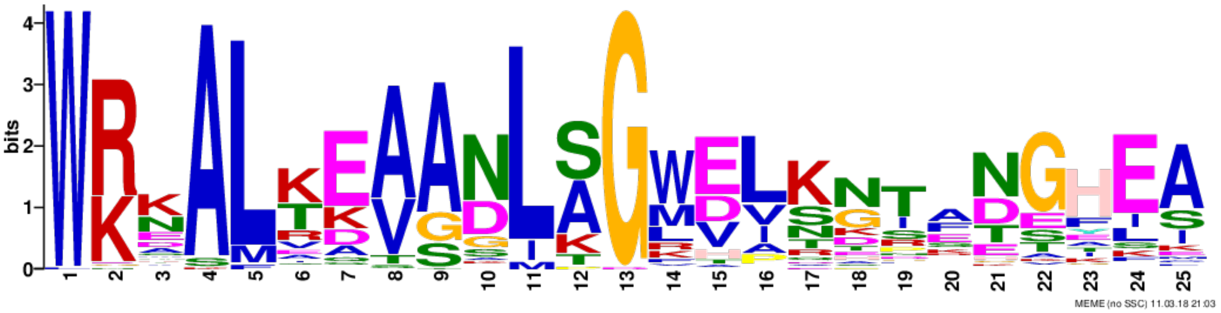

Motif 15:

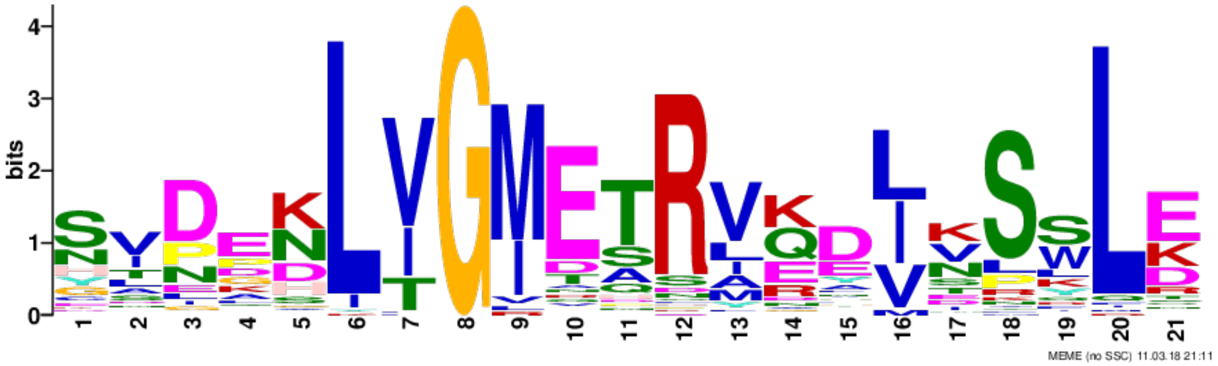

Motif 4: P loop

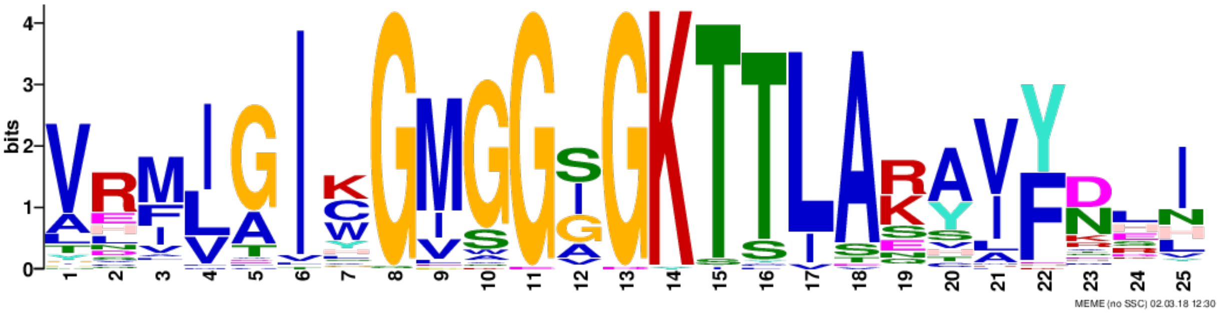

Motif 14:

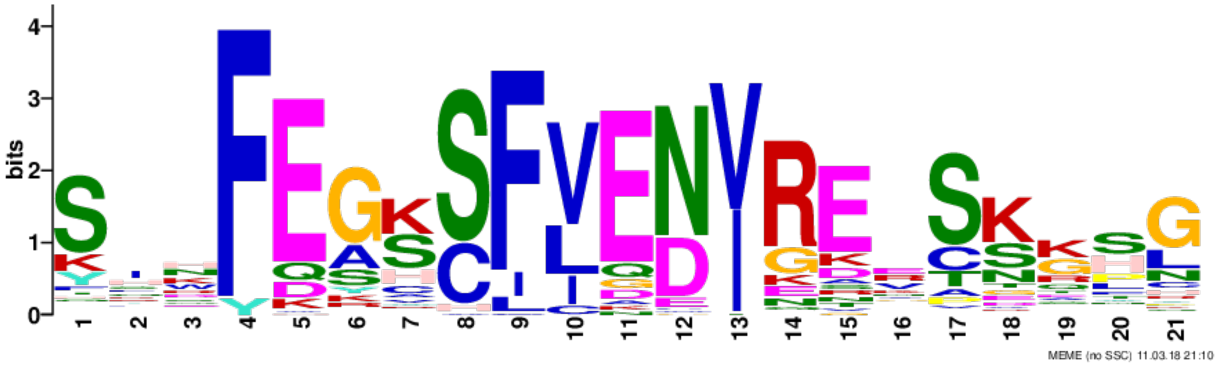

Motif 20: RNBS A

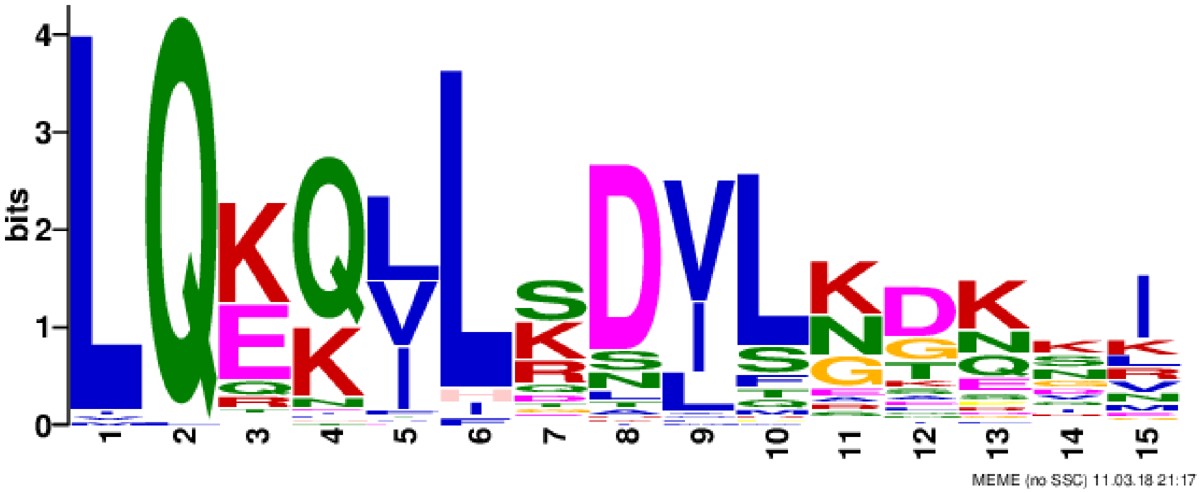

Motif 5: Kinase 2

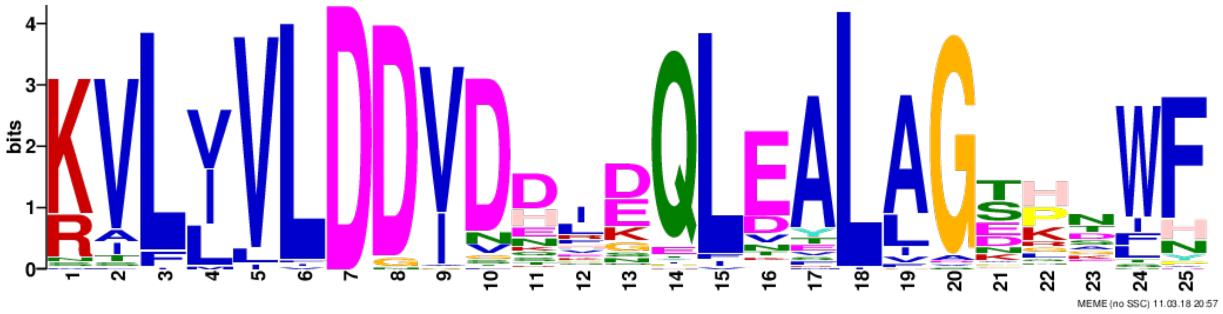

Motif 10: RNBS B

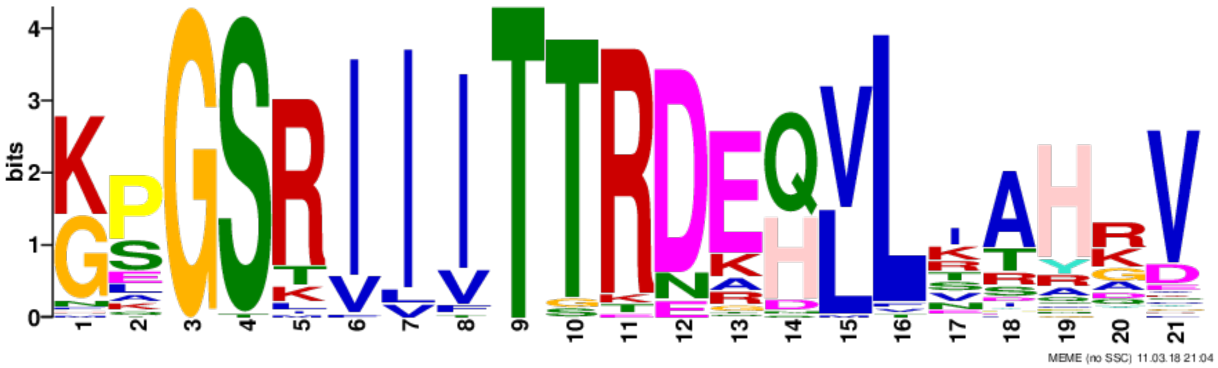

Motif 13: RNBS C

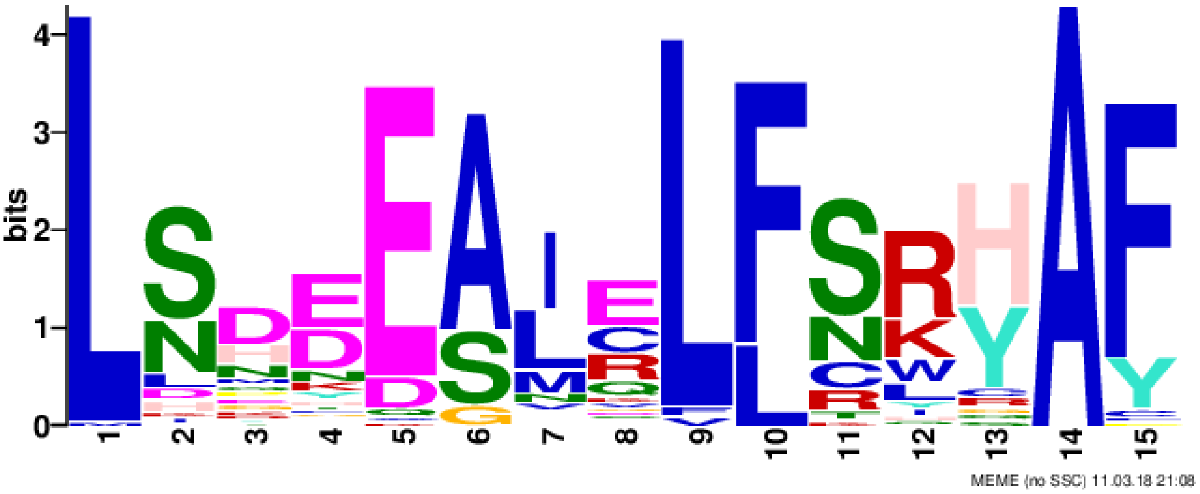

Motif 6: GLPL

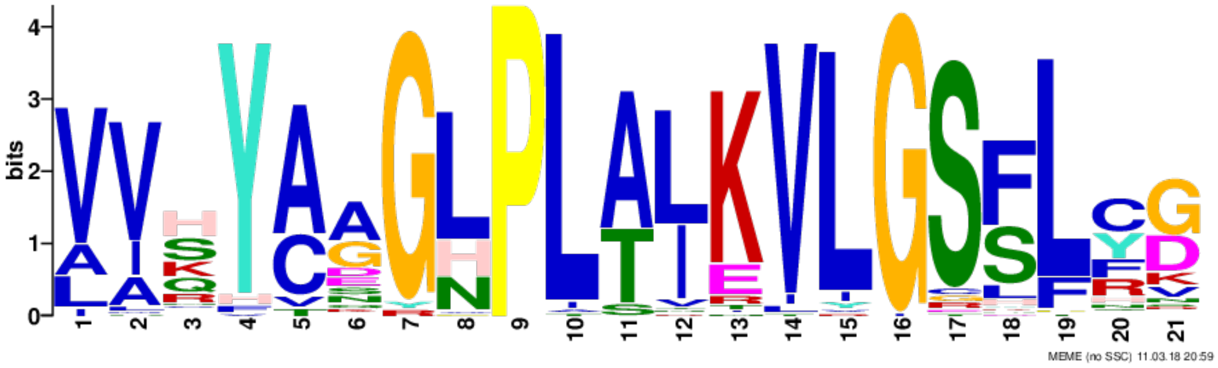

Motif 17: TNBSI

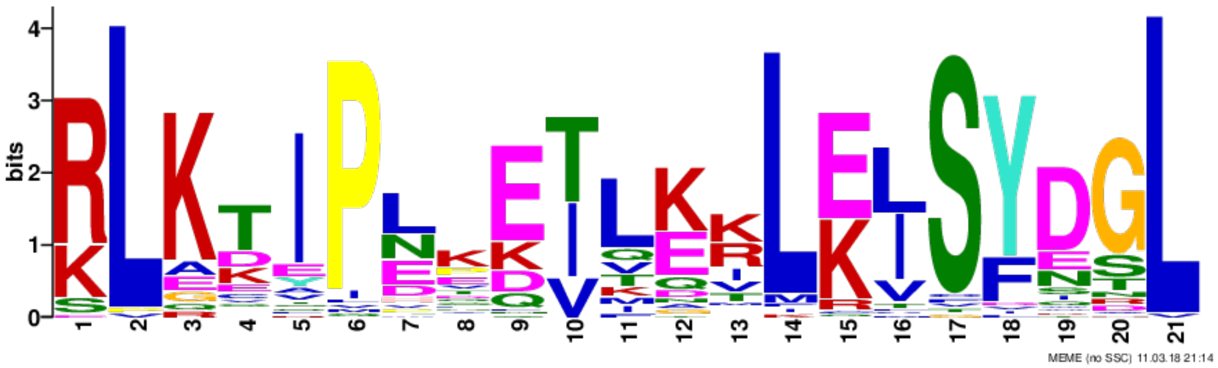

Motif 12: RNBS D

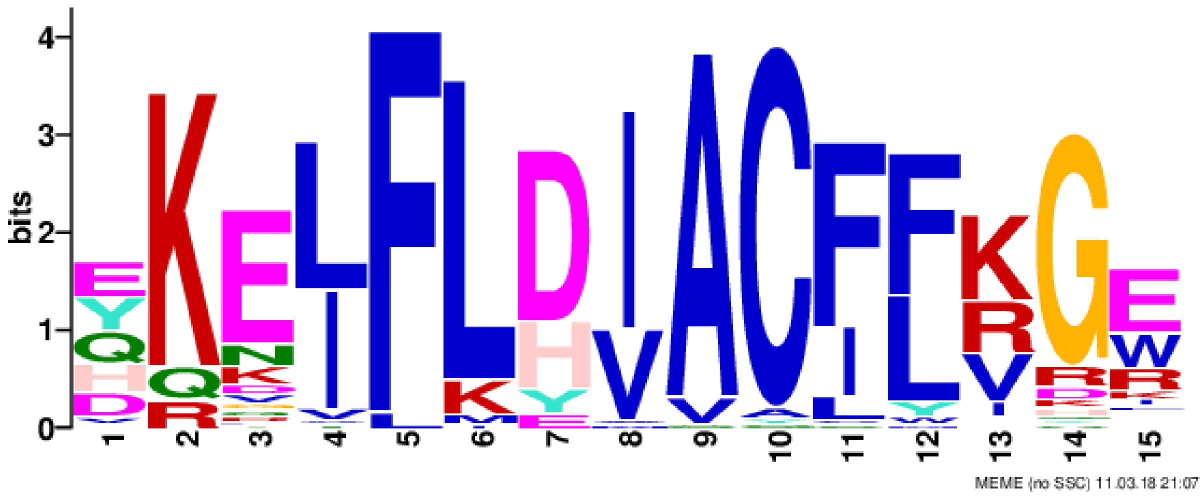

Motif 11:

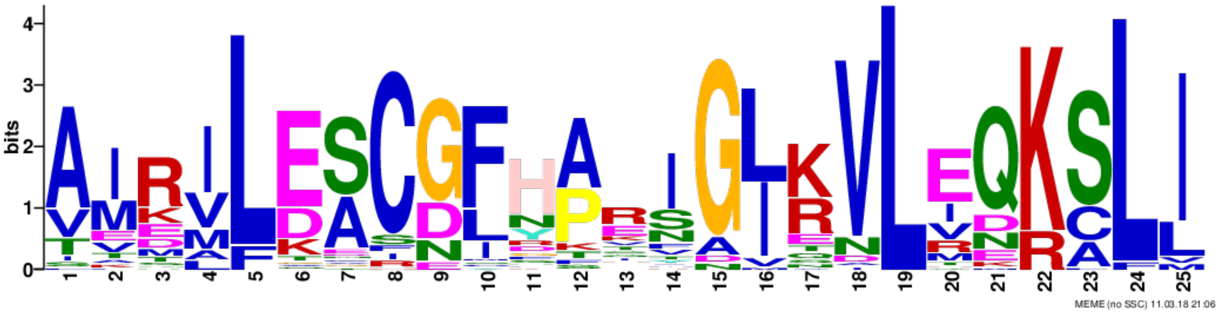

Motif 7: MHDL

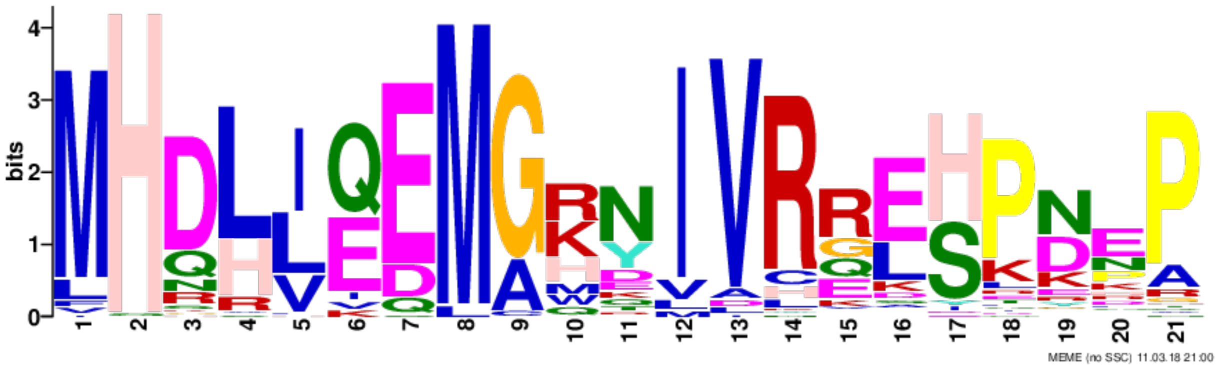

Motif 18: L1

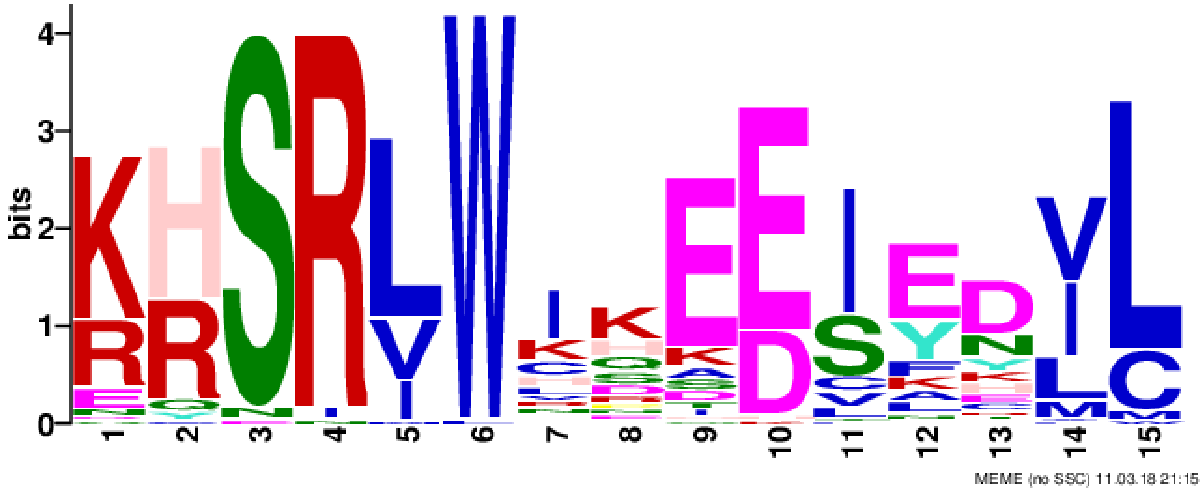

Motif 16:L2

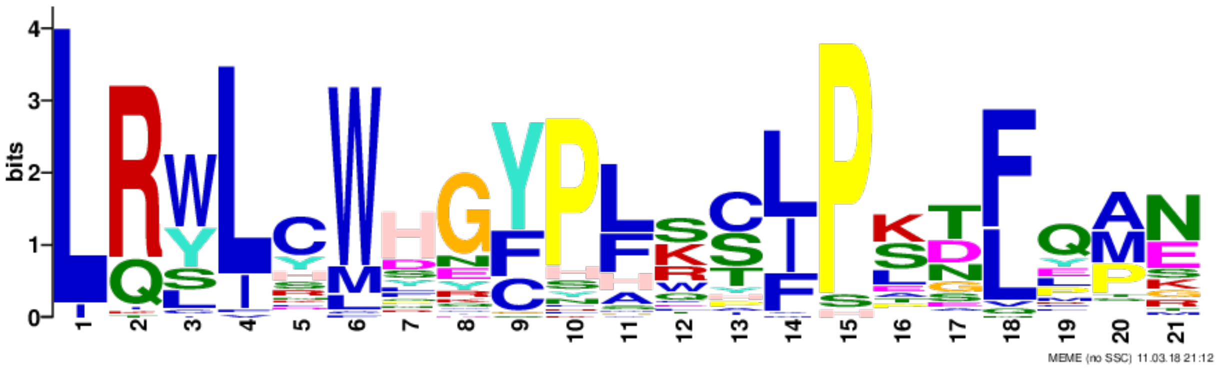

Supplementary File S1c. The motif sequence Logos in the sunflower RNL family of R proteins

Motif 19:RPW8-1

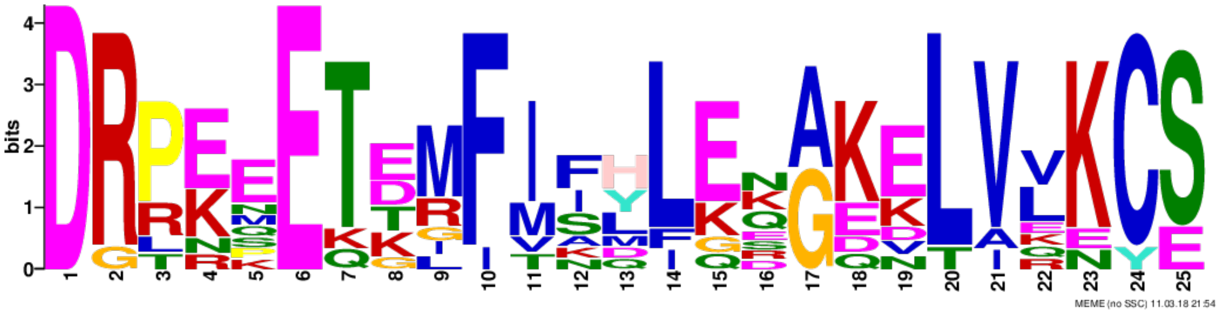

Motif 8:RPW8-2

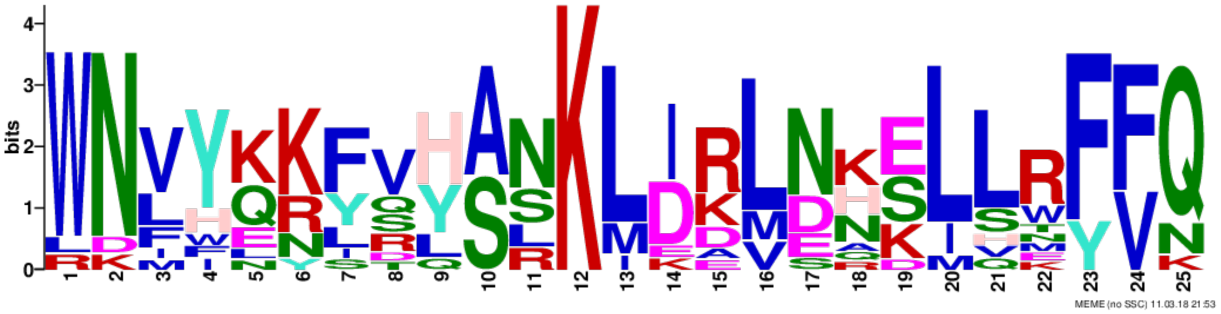

Motif 1: P-LOOP

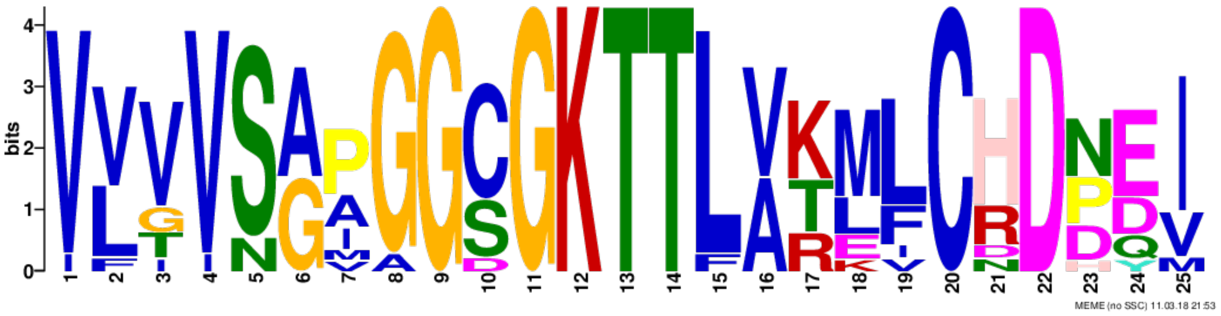

Motif 14: RNBS A

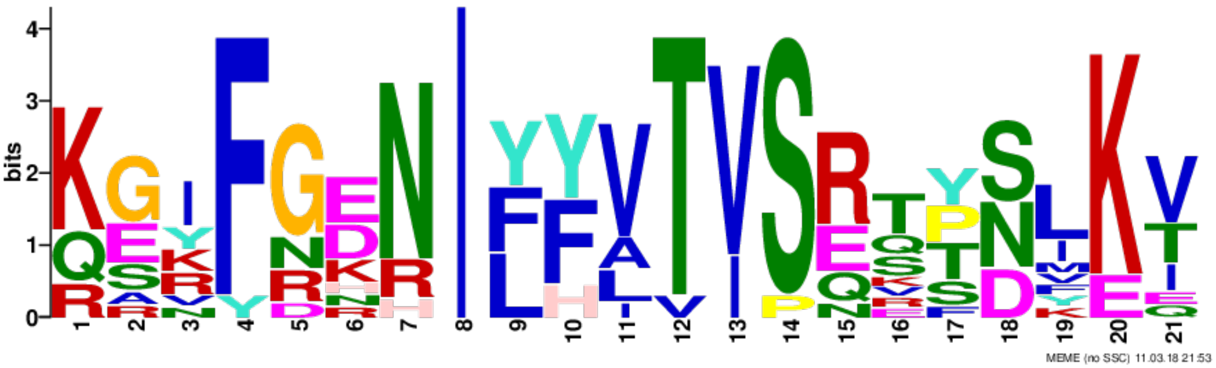

Motif 20:

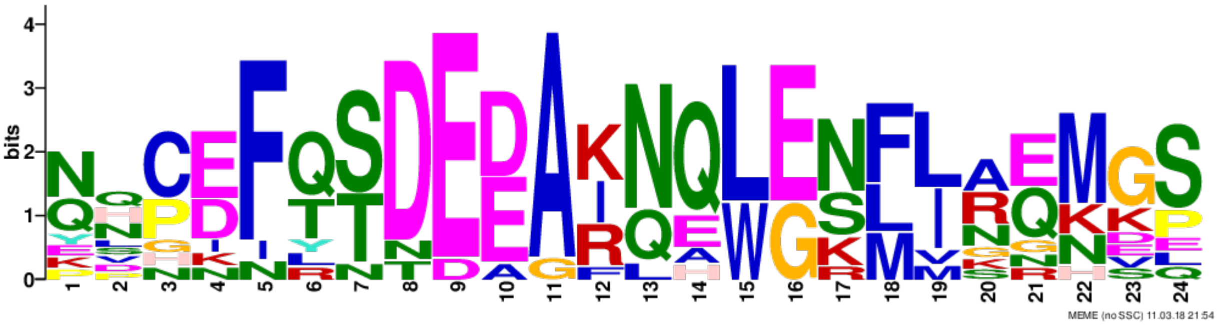

Motif 18: Kinase 2

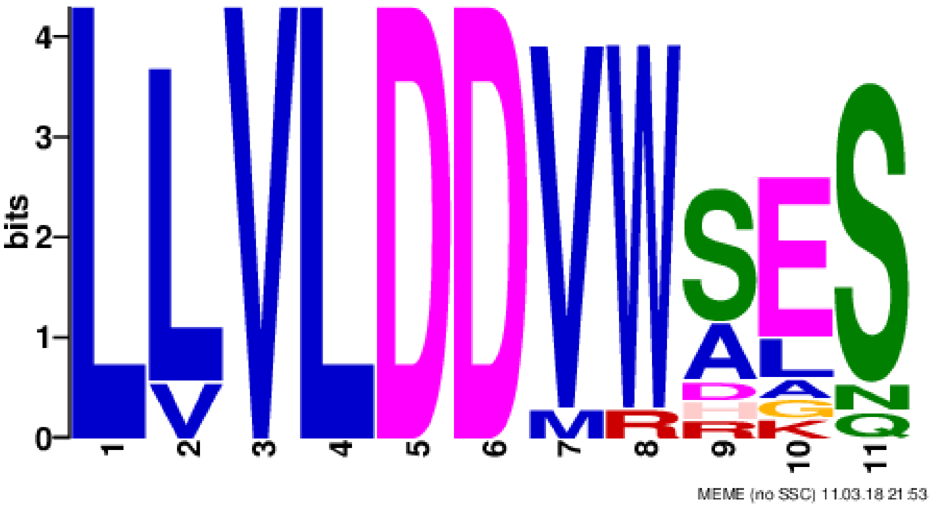

Motif 5: RNBS B

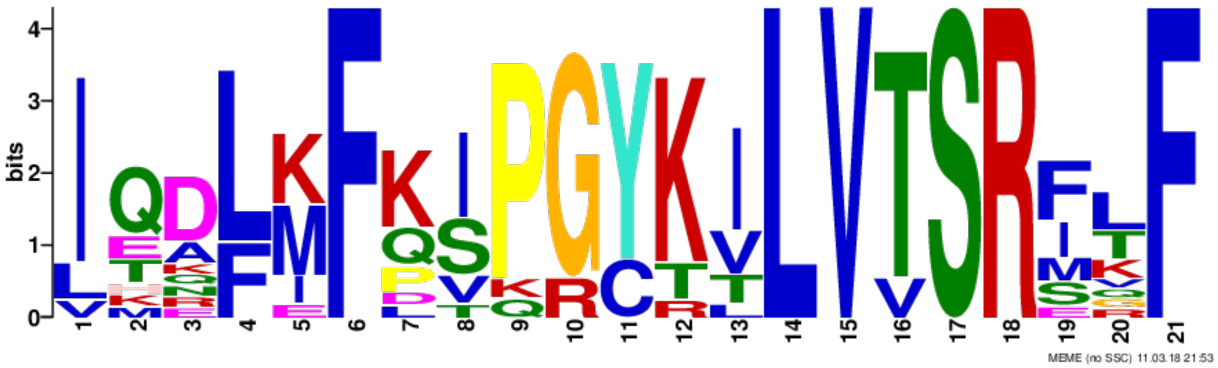

Motif 6: RNBS C

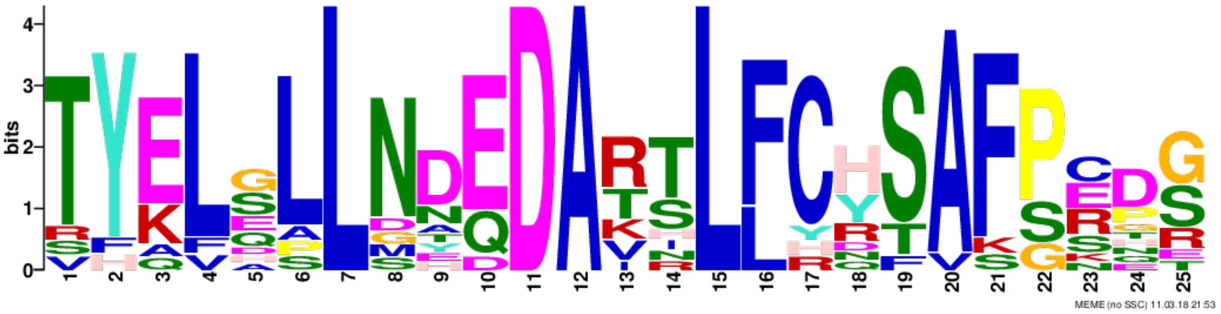

Motif 3:GLPL

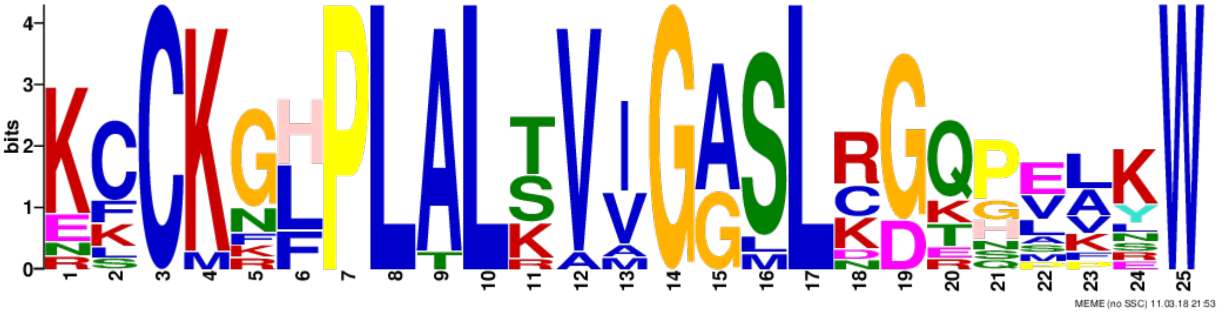

Motif 2:RNBS D

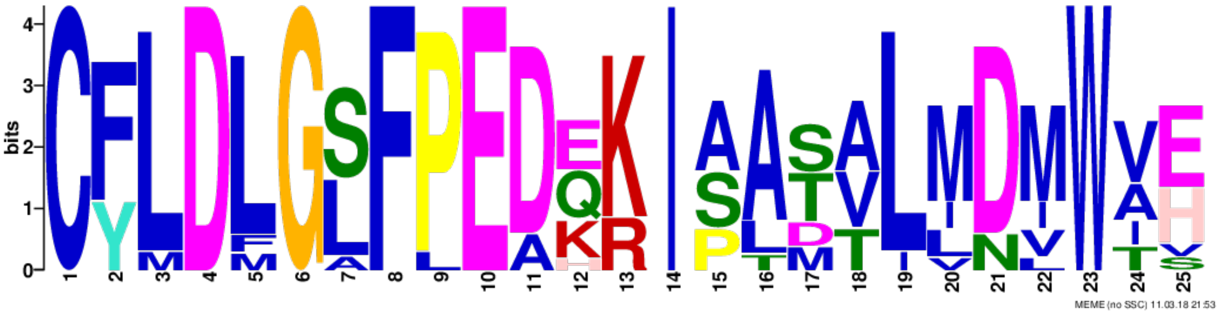

Motif 16:

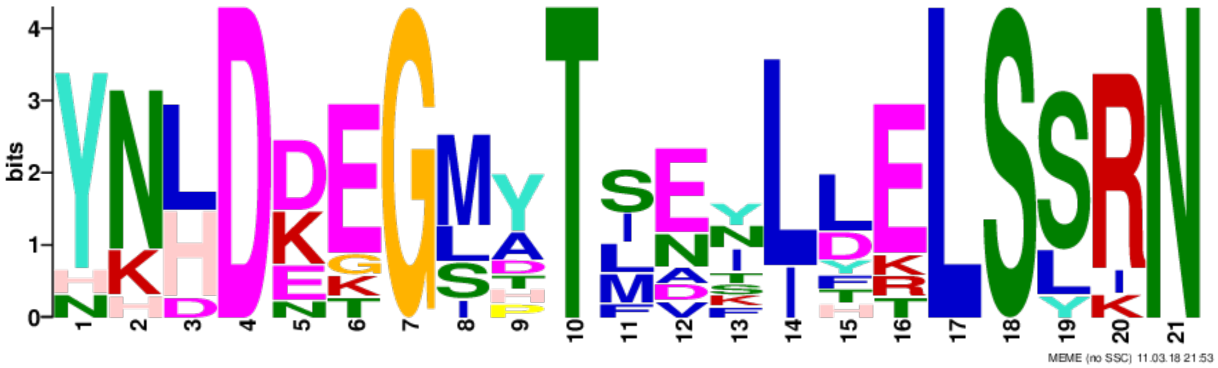

Motif 4: QHDL

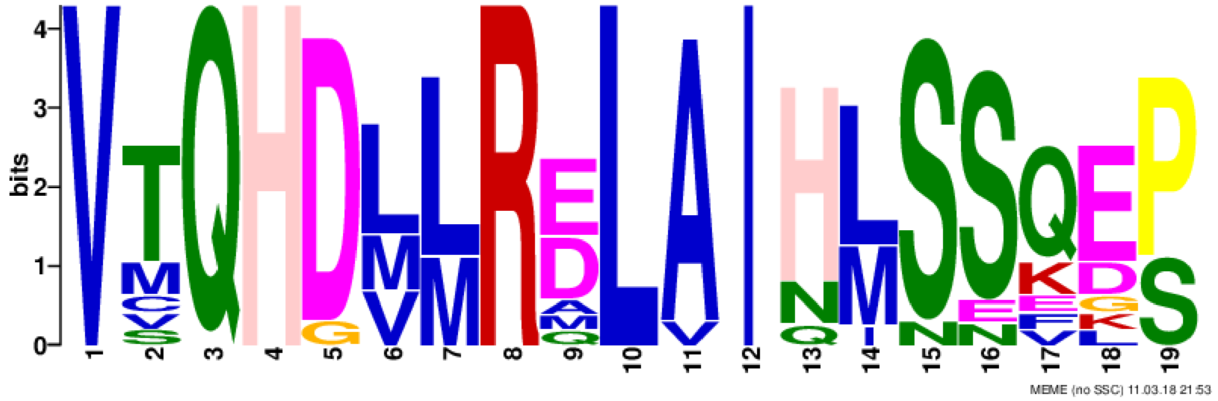

Motif 10:

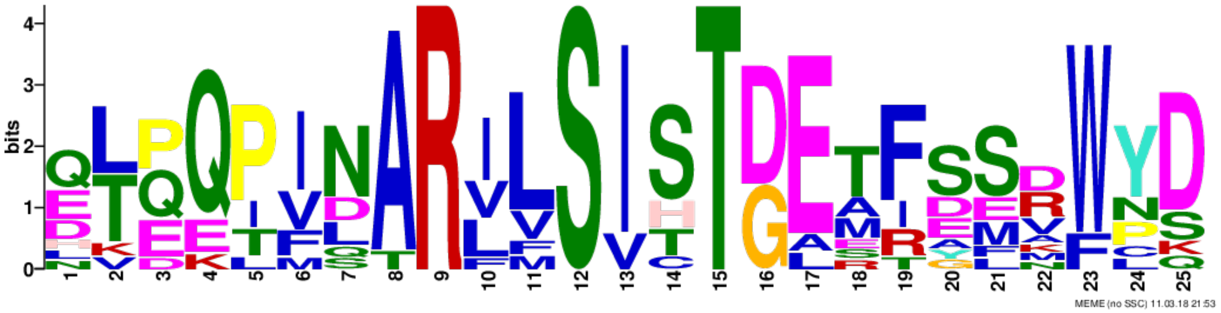

Motif 7:

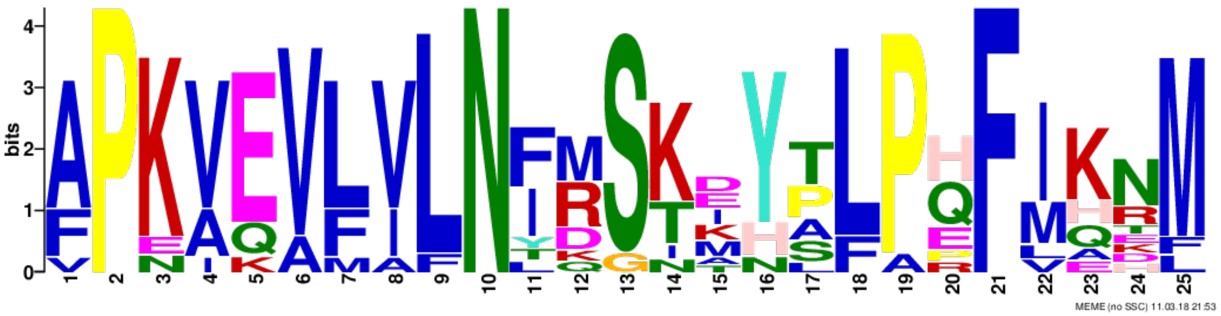

Motif 12: L1

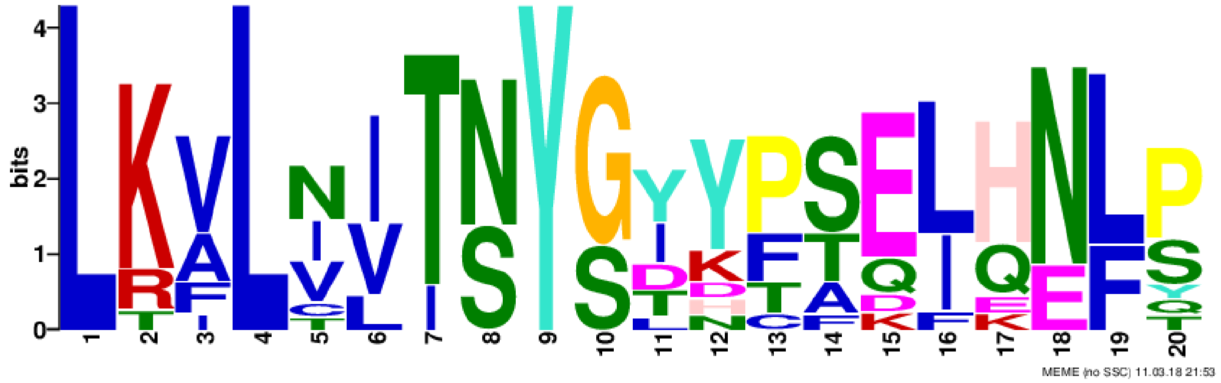

Motif 17:L2

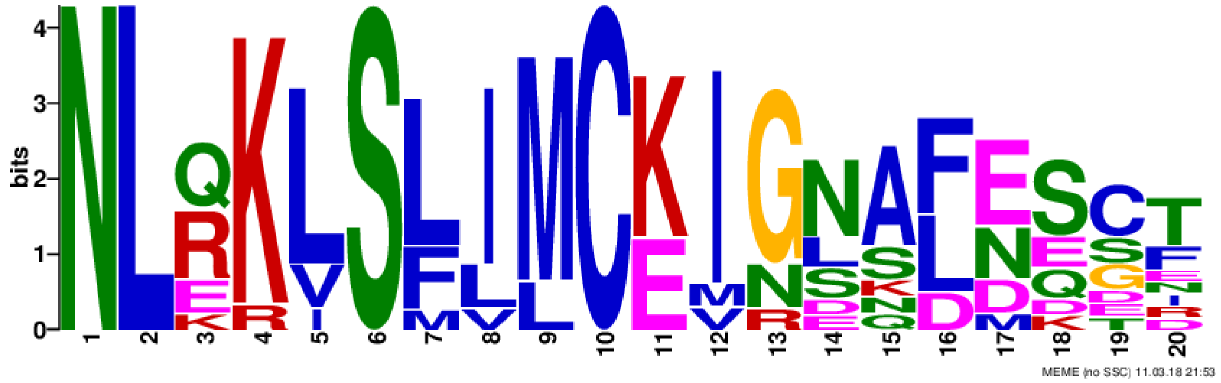

Motif 9:L3

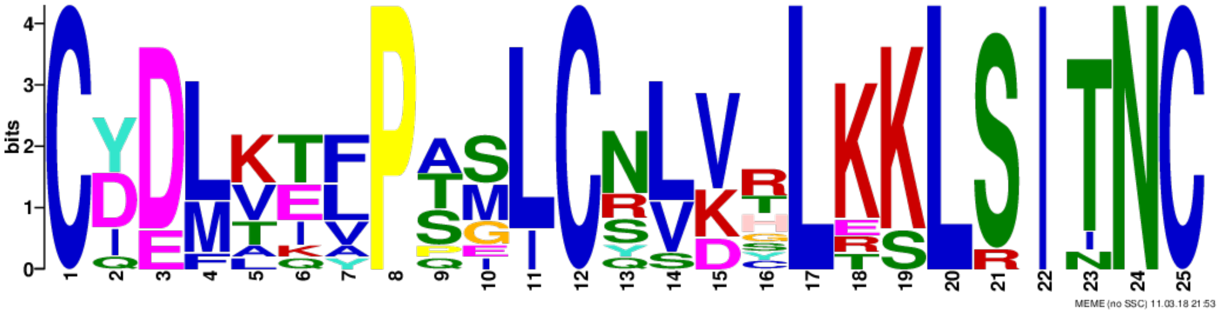

Motif 11:L4

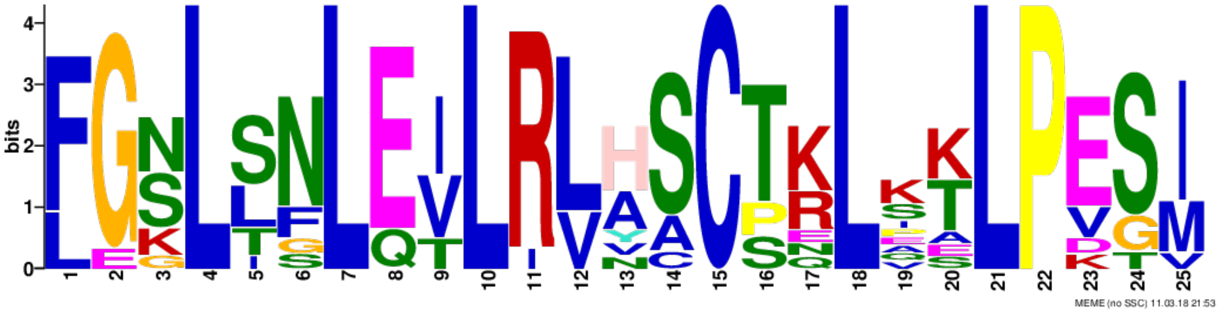

Motif 13: L5

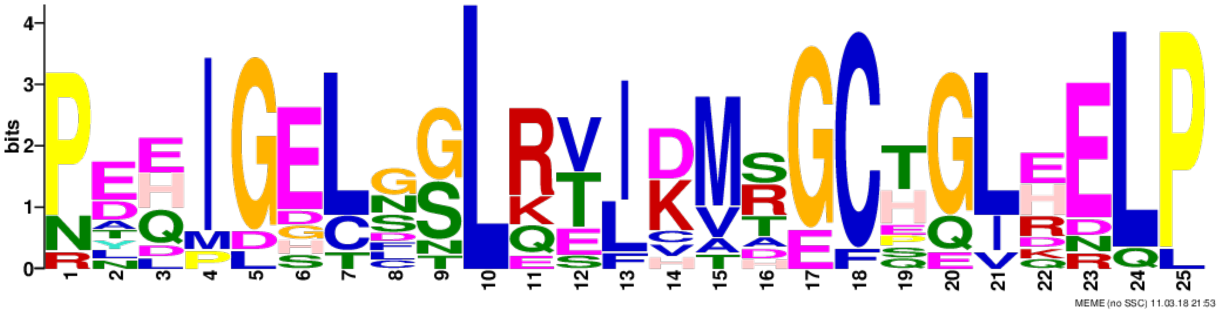

Motif 15:

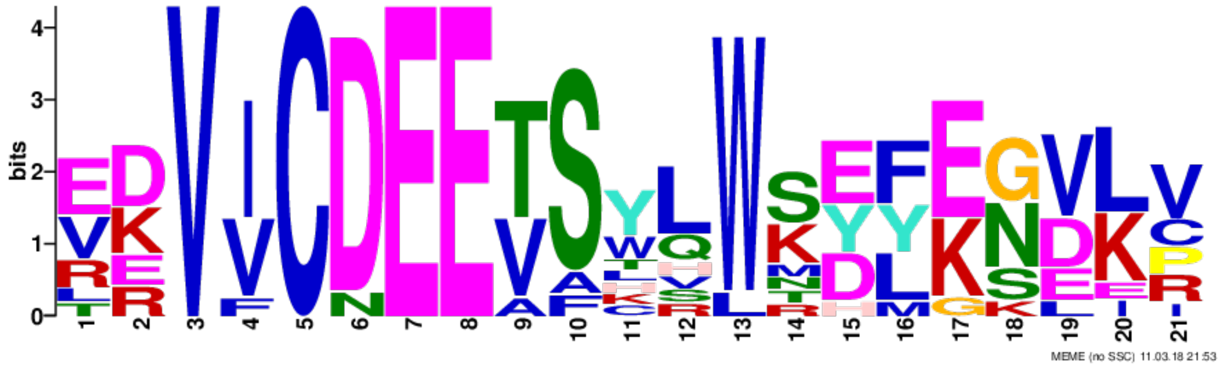

Supplement: Supplementary file 1 [file genes-09-00384-s001.zip › Supplementary Files/Supplementary File S1.pdf]
