# Supplementary figures and images for: Genome-Wide Identification of NBS-Encoding Resistance Genes in Sunflower (Helianthus annuus L.)
Source: Genes (Basel). 2018 Jul 30;9(8):384. doi: 10.3390/genes9080384 (PMC6115920; doi:10.3390/genes9080384)

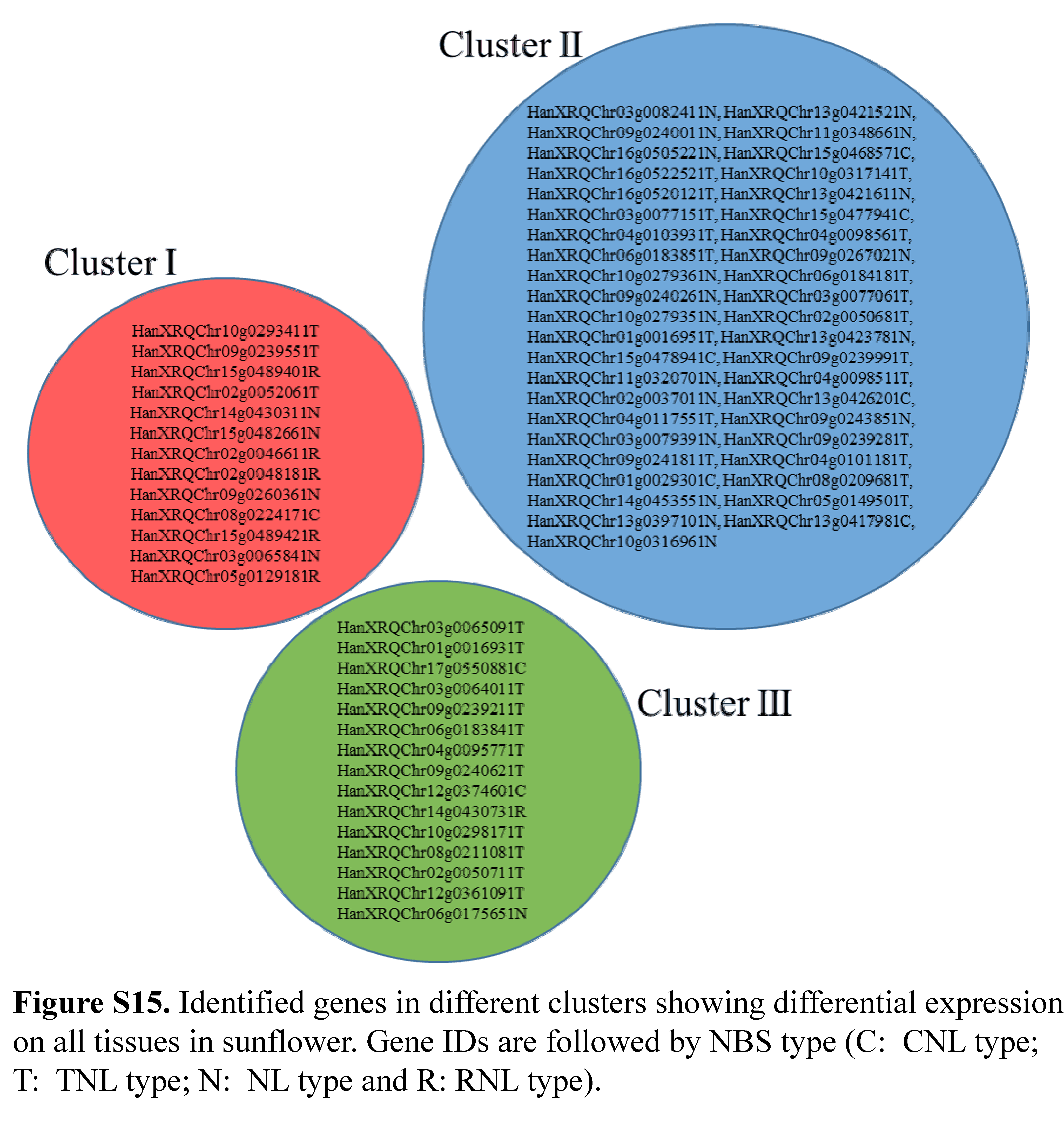

Supplement: Supplementary file 1 [file genes-09-00384-s001.zip › Supplementary Files/Figure S15.tif]

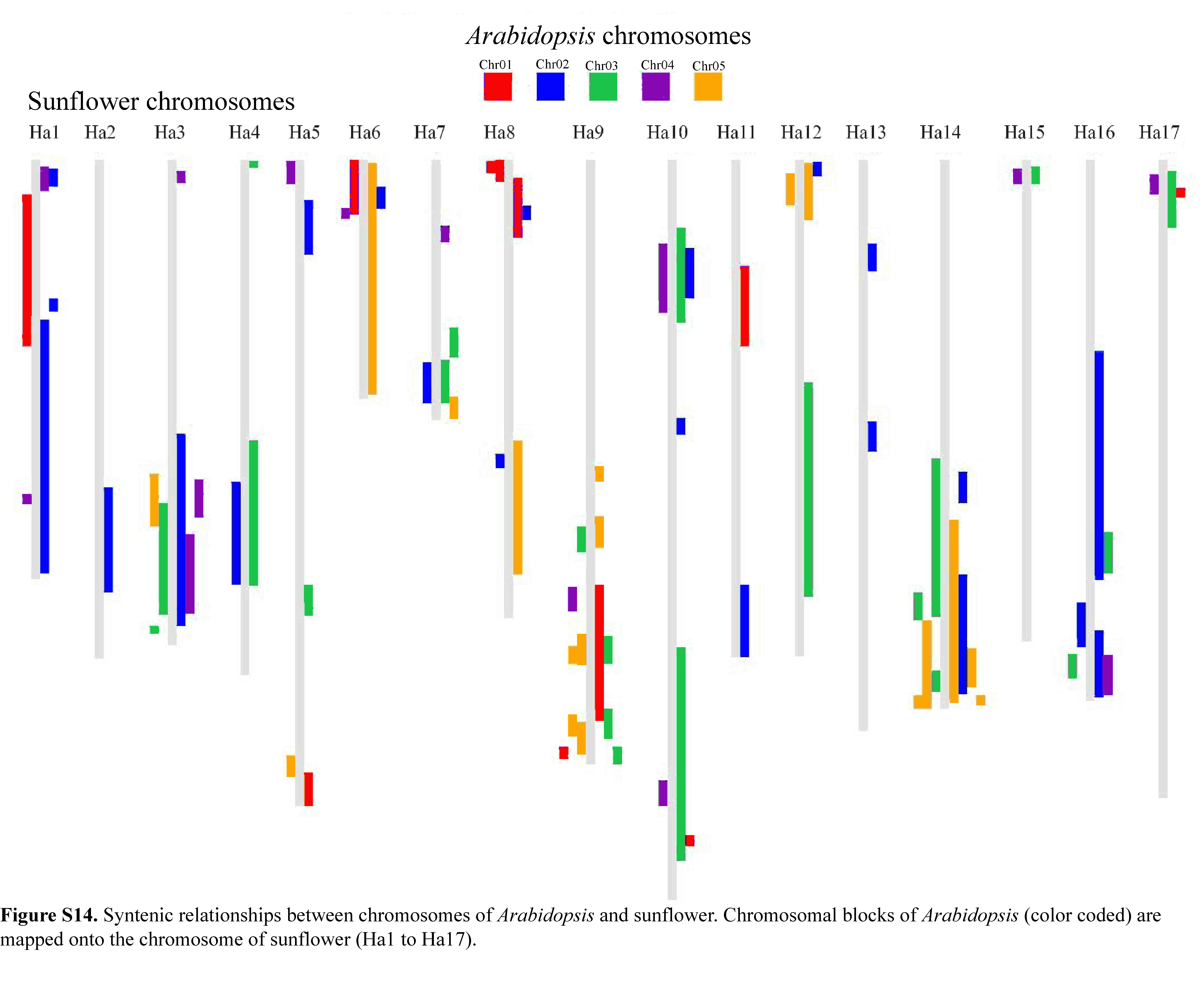

Supplement: Supplementary file 1 [file genes-09-00384-s001.zip › Supplementary Files/Figure S14.tif]

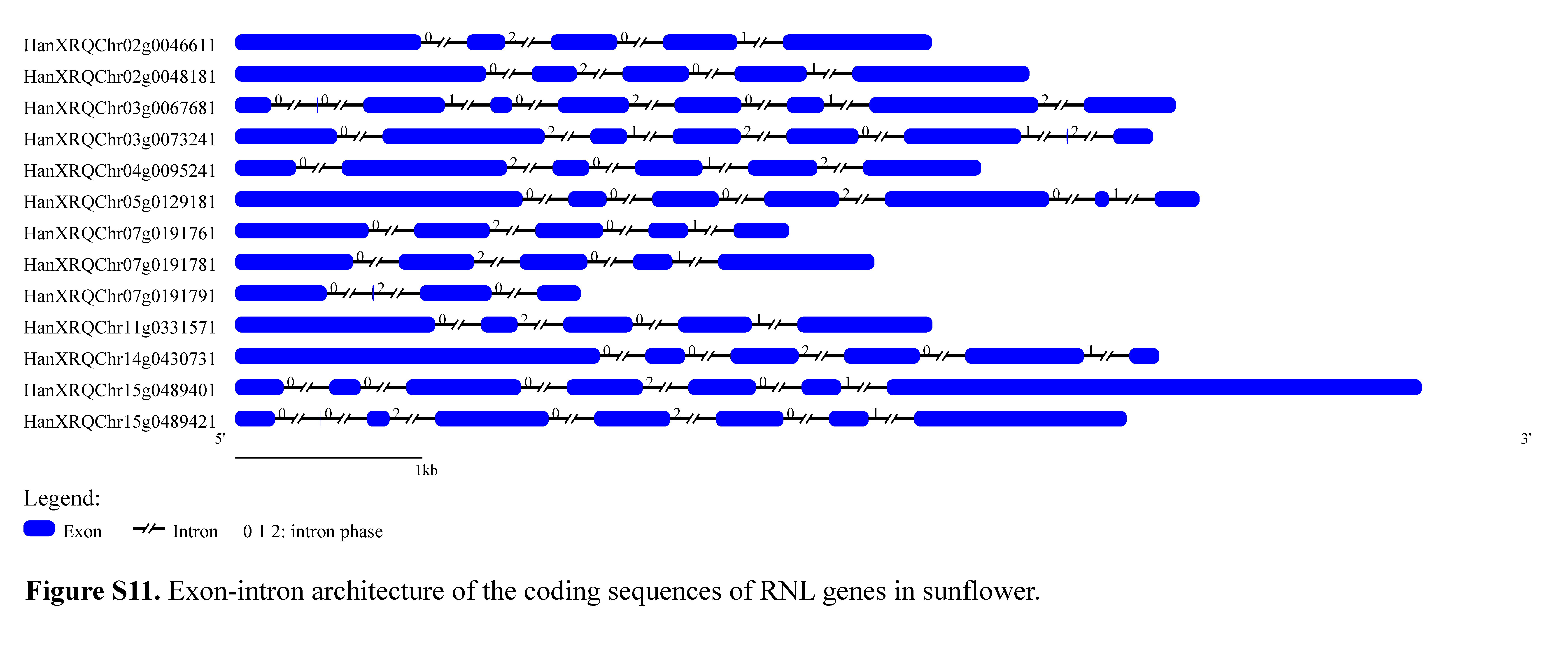

Supplement: Supplementary file 1 [file genes-09-00384-s001.zip › Supplementary Files/Figure S11.tif]

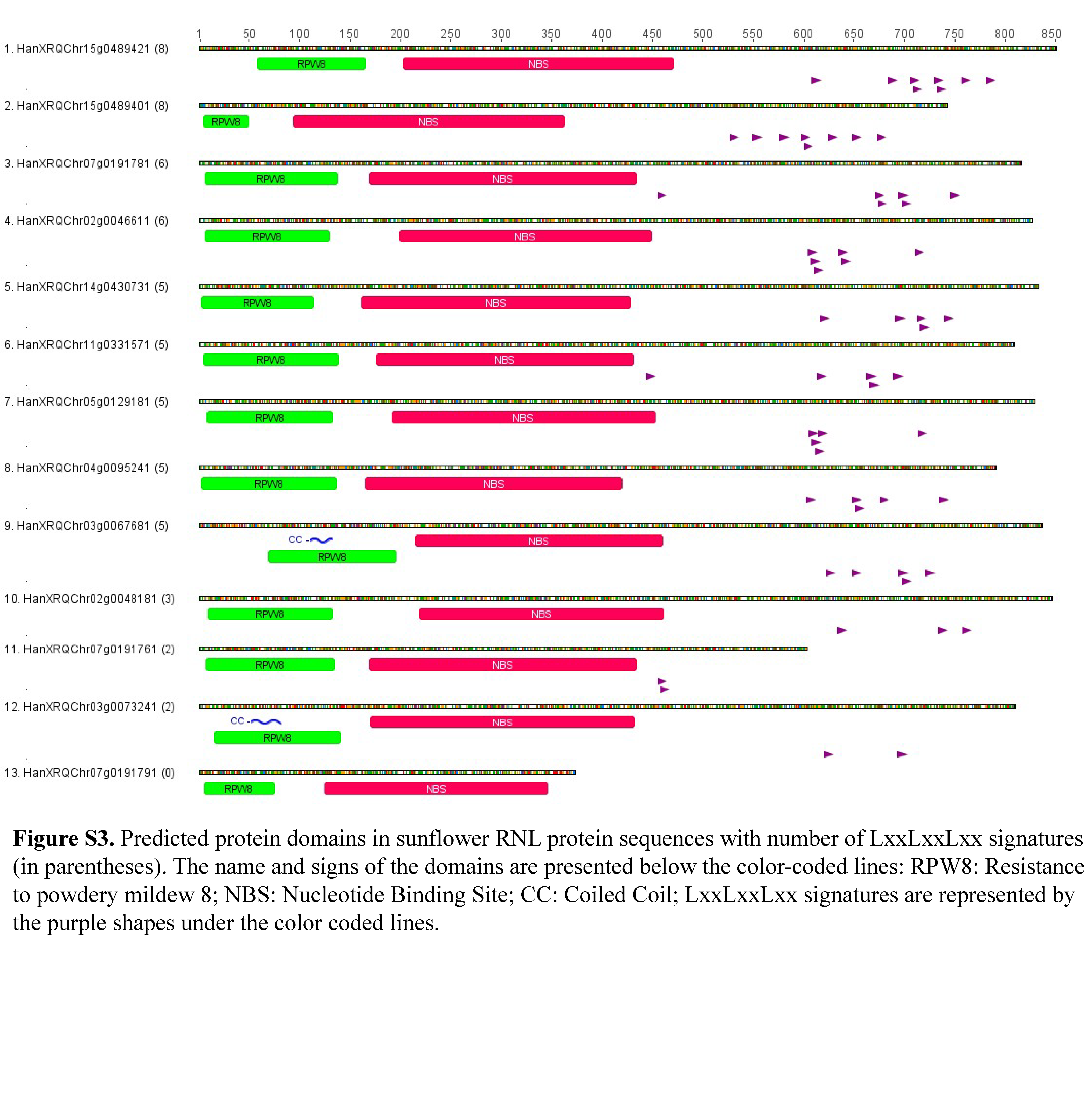

Supplement: Supplementary file 1 [file genes-09-00384-s001.zip › Supplementary Files/Figure S3.tif]

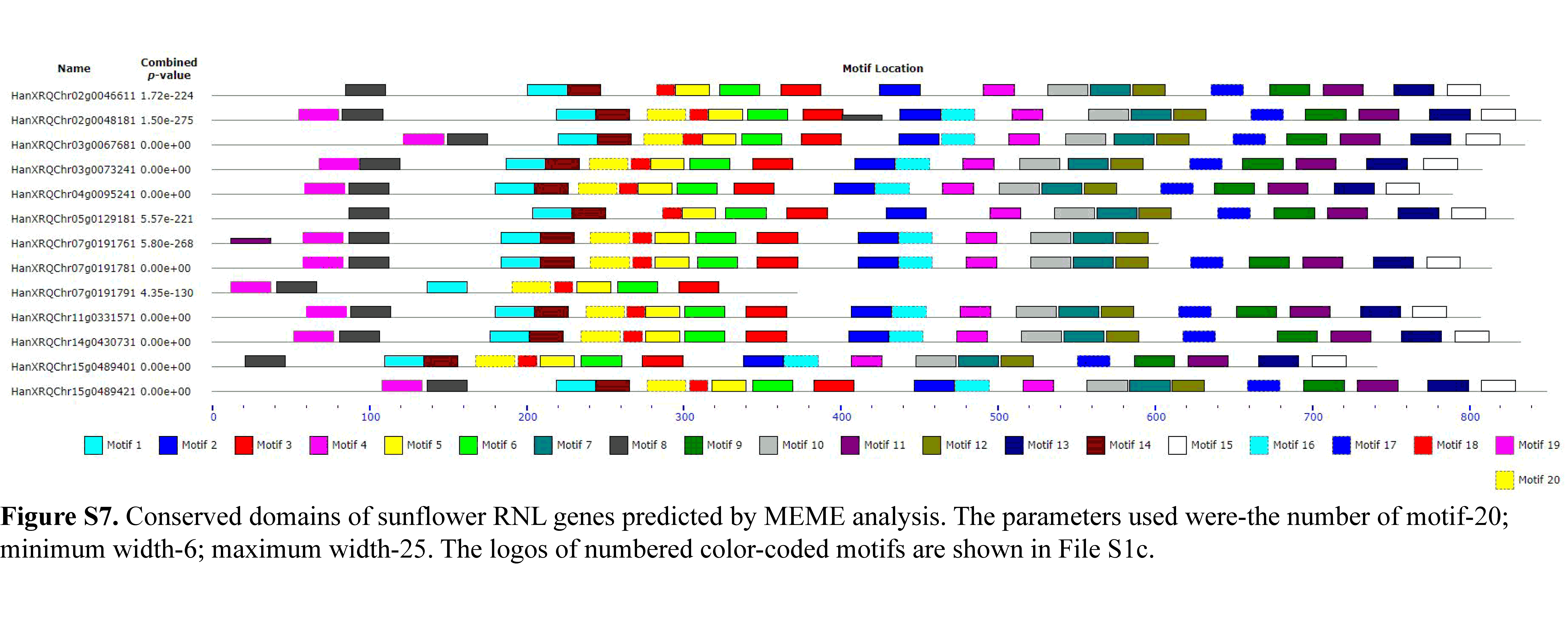

Supplement: Supplementary file 1 [file genes-09-00384-s001.zip › Supplementary Files/Figure S7.tif]

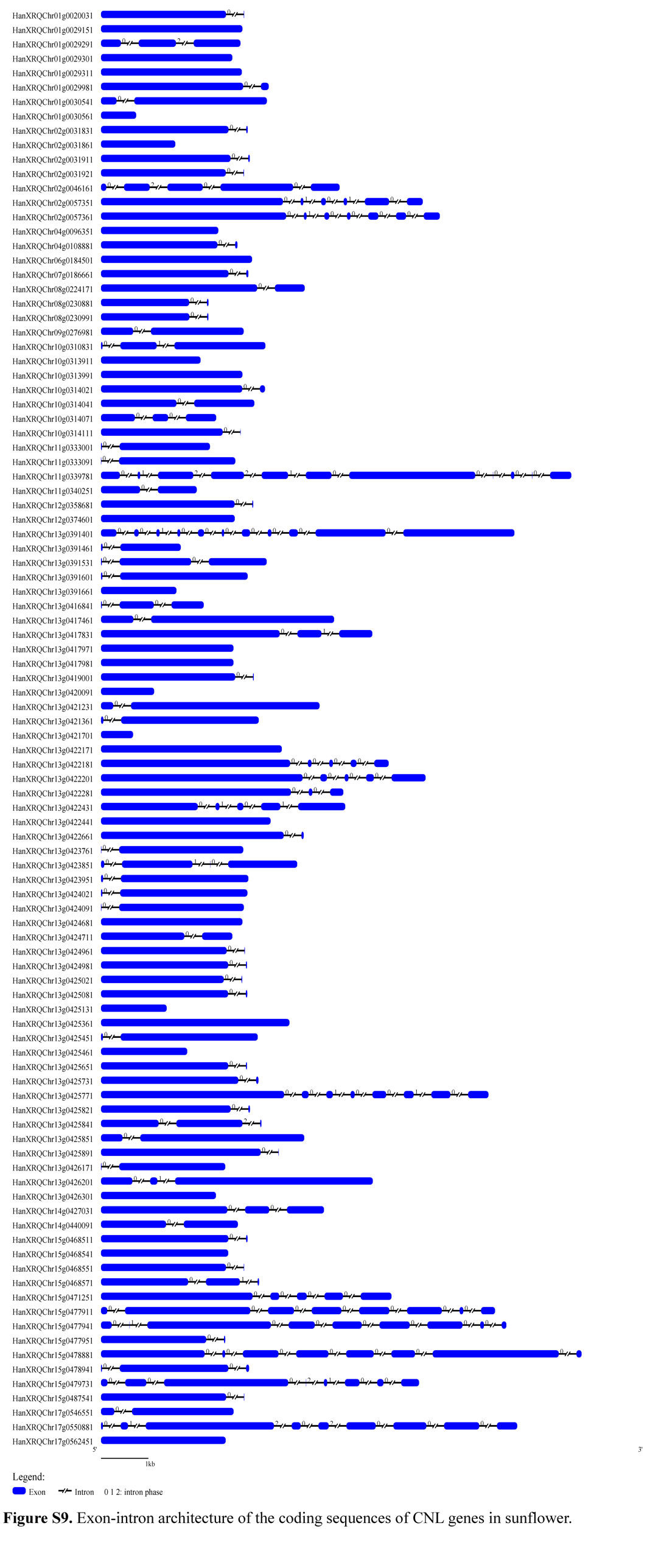

Supplement: Supplementary file 1 [file genes-09-00384-s001.zip › Supplementary Files/Figure S9.tif]
