# Supplementary material for: Genome-Wide Identification of NBS-Encoding Resistance Genes in Sunflower (Helianthus annuus L.)
Source: Genes (Basel). 2018 Jul 30;9(8):384. doi: 10.3390/genes9080384 (PMC6115920; doi:10.3390/genes9080384)
Supplement: Supplementary file 1 [file Figure S4.pdf › Supplementary Files/Figure S4.pdf]

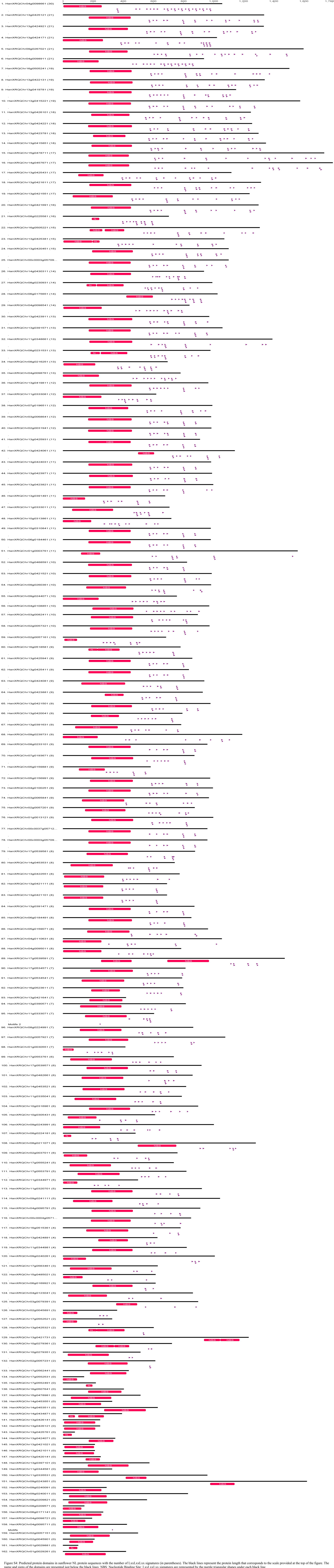

Figure S4: Predicted protein domains in sunflower NL protein sequences with the number of LxxLxxLxx signatures (in parentheses). The black lines represent the protein length that corresponds to the scale provided at the top of the figure. The name and signs of the domains are presented just below the black lines: NBS: Nucleotide Binding Site; LxxLxxLxx signatures are represented by the purple triangular shapes under each black line.
