# Supplementary material for: Genome-Wide Identification of NBS-Encoding Resistance Genes in Sunflower (Helianthus annuus L.)
Source: Genes (Basel). 2018 Jul 30;9(8):384. doi: 10.3390/genes9080384 (PMC6115920; doi:10.3390/genes9080384)
Supplement: Supplementary file 1 [file Figure S1.pdf › Supplementary Files/Figure S1.pdf]

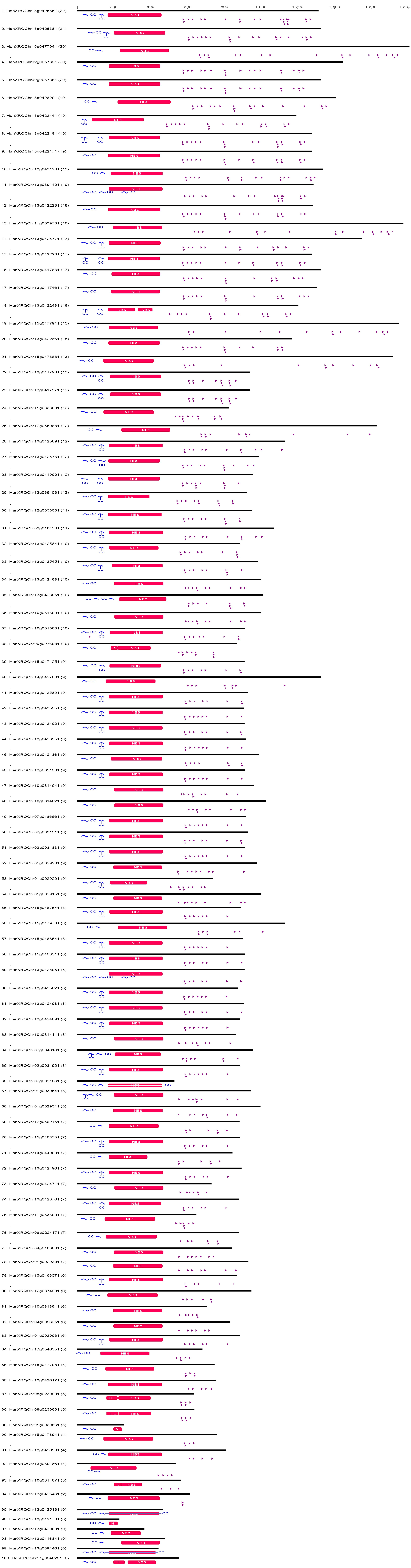

Figure S1. Predicted protein domains in sunflower CNL protein sequences with number of LxxLxxLxx signatures in parentheses. Each black line represents amino acid sequence lengths that correspond to the scale provided at the top of the figure. The name and signs of the domains are presented below the black line: CC: Coiled Coil; NBS: Nucleotide Binding Site; RX_CC: Coiled coil domain present in disease resistance protein, Rx; LxxLxxLxx signatures are represented by the purple shapes under the black lines.
